# The associations between leadership styles and perceived insider status: a meta-analysis

**DOI:** 10.3389/fpsyg.2025.1631075

**Published:** 2025-11-13

**Authors:** Yufan Lin, Yingkai Yang, Yimo Shen

**Affiliations:** Faculty of Psychology, Southwest University, Chongqing, China

**Keywords:** perceived insider status, leadership styles, inclusive leadership, meta-analysis, relative weight analysis

## Abstract

The perceived insider status of employees is crucial for the development of both organizations and individuals. This paper provides the first meta-analytic examination of the relationships between leadership styles and followers’ perceived insider status. The meta-analysis examined 12 leadership styles across 137 articles, comprising a total of 151 effect sizes (*N* = 45,228). The results revealed significant positive correlations between leader-member exchange, differential leadership, inclusive leadership, participative leadership, transformational leadership, empowering leadership, authentic leadership, servant leadership, humble leadership, benevolent leadership, moral leadership, and perceived insider status. Conversely, authoritarian leadership showed a significant negative correlation with perceived insider status. Additionally, the results of relative weight analysis indicated that inclusive leadership exhibited the strongest explanatory power for perceived insider status, while transformational leadership showed the weakest explanatory power. Furthermore, moderation analysis revealed that there were no significant moderation effects of study design, leadership measurement tools, publication status, gender, or age on the relationship between leadership and perceived insider status.

## Introduction

The concepts of “insiders” and “outsiders” have long been prevalent in the field of organizational management (Stamper and Masterson, 2002; Wang and Kim, 2013), traditional distinctions between “insiders” and “outsiders” often focus on objective classifications, such as the criticality of employees to organizational operations (Tsui et al., 1995), tenure within the organization, average working hours, and so forth. The most typical groups of insiders and outsiders in an organization are full-time employees and part-time employees (Pfeffer and Baron, 1988). Research has shown that compared to part-time employees, full-time employees tend to express more prosocial tendencies (Kassing et al., 2018), report higher level of organizational commitment in the workplace (Marchese and Ryan, 2001). However, whether the differential organizational outcomes resulting from insiders and outsiders are due to actual differences in employee status or employees’ perceptions of their insider or outsider status? To address this issue, Stamper and Masterson (2002), proposed the concept of perceived insider status based on the level of exchange between employees and the organization and demonstrated in their study that the actual inclusion of organizations does not lead to perceived insider status, their exist other factors that more important to employees’ perceived insider status.

Perceived insider status has been proven to be positively correlated with many beneficial outcomes. For organizations, perceived insider status can enhance employee job performance, innovative behaviours (Chen and Aryee, 2007) and facilitate employee voice (Liu et al., 2022), bringing about better returns for the organization. On an individual level, perceived insider status can satisfy employees’ growth needs, enhance their job satisfaction, and foster a sense of belonging (Zhao and Liu, 2020).

Consequently, an increasing number of scholars are focusing on how companies can enhance employees’ perceived insider status. Some Researchers have found that the quality of the exchange relationship between employees and leaders can significantly influence employees’ internal identity cognition (Stamper and Masterson, 2002). From the perspective of social identity theory, supervisors, as organizational agents, signal an employee’s value and belonging through their treatment. If employees receive incentives such as trust, support and empowerment from their supervisor, they will feel like they are part of the organization and gain the personal space (Wang et al., 2009). Moreover, as an authority, supervisor’s treatment of employees can influence their perception of inclusiveness and belonging (Lapalme et al., 2009), when employees perceive support from supervisor, they are more likely to believe they are one of their organizations and have in-group status (Hui et al., 2015). Demonstrating that leadership is one of the important influencing factors of employees’ perceived insider status.

A review of the extant literature reveals a complex and inconsistent landscape regarding the relationships between specific leadership styles and perceived insider status. For example, Servant leadership has been found to promote employees’ perceived insider status (Yeh et al., 2022), while authoritarian leadership tends to decrease it (Schaubroeck et al., 2017). And even within the same leadership style, there may be varying degrees of correlation with perceived insider status. For instance, research indicates a negative correlation between authoritarian leadership and perceived insider status (Schaubroeck et al., 2017), but other studies have found a positive correlation between the two (Zhang et al., 2021). The correlation coefficient between differential leadership style and perceived insider status ranges from 0.14 (Zhang et al., 2022) to 0.84 (Li et al., 2019). Therefore, questions arise about the exact nature of the relationship between leadership styles and perceived insider status, the differences in interpretations of perceived insider status, and the factors influencing these relationships. To address these questions, it becomes necessary to conduct a meta-analysis of the relationship between leadership styles and perceived insider status.

Despite the abundance of primary studies, a systematic, quantitative synthesis of this body of research is absent. This literature gap limits our ability to draw definitive conclusions about the overall strength of the relationship between leadership and perceived insider status and the sources of its variability. Therefore, this article compiles both Chinese and English literature on leadership styles and perceived insider status. Using meta-analytic methods, it discusses the influence of leadership styles on perceived insider status and compares the explanatory power of different leadership styles on perceived insider status. Additionally, it considers the effects of different leadership measurement tools, study designs, publication status, gender, and age on the relationship between leadership styles and perceived insider status. By doing so, this meta-analysis seeks to consolidate existing findings and offer evidence-based recommendations for organizational practice. Theory and Hypotheses.

### Conceptualizing perceived insider status

The concept of perceived insider status was initially proposed by Stamper and Masterson (2002), reflecting the extent to which employees perceive themselves as “insiders” within an organization. And also representing the feeling of organizational members about their personal space and recognition within the organization, primarily measuring employees’ sense of belonging in the organization (Masterson and Stamper, 2003). According to inducement-contribution model (March and Simon, 1958), employers trade inducements for employees’ contributions, the more meaningful the inducement, the more employees will contribute. Subsequently, scholar proposed the inducements and contributions theory (Hipple, 1998), compared to part-time employees, full-time employees are provided with more opportunities and benefits from the organization, leading to a sense of obligation to contribute to the organization. This inducement-contribution cycle prompts some employees to develop a greater sense of dedication to the organization, thus distinguishing between insiders and outsiders (Stamper and Masterson, 2002). The concept of insiders and outsiders is similar to that of in-group and out-group which originated from leader-member exchange. In the theory of leader-member exchange, supervisors will treat subordinates as member of in-group or out-group based on their relationship, while Stamper and Masterson (2002) proposed that employees can perceive themselves as insiders or outsiders according to the differential relationships with the organization. Despite the similarities with Leader-Member Exchange, perceived insider status still has its own uniqueness. First, LMX represents a dyadic phenomenon between leader and employee, while PIS is about the perceived relationship between an employee and an organization. Second, LMX is a measure of the employee-supervisor relationship, but PIS measures the feelings of insider status in the organization.

In subsequent research, some scholars have proposed that perceived insider status is one of the important dimensions of employees’ self-concept (Chen and Aryee, 2007), serving as a channel through which individuals perceive themselves as making positive contributions to the workplace (Kim et al., 2009). In the context of China, perceived insider status is also regarded as employees’ recognition of their “in-group” identity (Wang et al., 2009).

Numerous studies have explored the antecedents and outcome variables of perceived insider status. The most representative measurement tool is the six-item scale developed by Stamper and Masterson (2002), using a sample of 350 employees from six hotels in the United States. The scale consists of three positively scored items and three negatively scored items, with representative items such as “I strongly feel like I am a part of the organization.” This scale has been proven to have good reliability and validity not only in foreign contexts but also in research conducted in the Chinese context (Chen and Aryee, 2007).

### Conceptualizing leadership styles

Leadership is a composite of leader behaviours, characterized by consistent patterns of behaviour and traits (Ding et al., 2020). This study focuses on leadership styles that have been extensively explored in research on perceived insider status, including Leader-Member Exchange, Differential Leadership, Inclusive Leadership, Participative Leadership, Transformational Leadership, Empowering Leadership, Authentic Leadership, Servant Leadership, Humble Leadership, Authoritarian Leadership, Benevolent Leadership, and Moral Leadership (see Supplementary Table S1).

The Leader-Member Exchange (LMX) theory posits that leaders form different quality relationships with each subordinate by engaging in various types of social exchanges (Graen and Cashman, 1975). The commonly used measurement scale is the 7-item unidimensional LMX scale developed by Graen and Uhl-Bien (1995). Later, Liden and Maslyn (1998) divided LMX into four dimensions: affect, loyalty, contribution, and professional respect. Wang et al. (2004) revised a 16-item Chinese version based on this. Similar to LMX, Differential Leadership is another leadership style with Chinese characteristics proposed in the context of relationship differentiation. Taiwanese scholar Cheng (1995) proposed differential leadership theory, suggests that leaders in Chinese organizations differentiate “insiders” and “outsiders” among subordinates based on three criteria: affection, loyalty, and ability, and treat insiders preferentially. Jiang and Chang (2010) divided differential leadership into three dimensions: communication care, tolerance trust, and promotion reward, and developed a 14-item scale. Inclusive Leadership, also a relationship-oriented leadership style, involves leaders listening to subordinates’ perspectives, welcoming and encouraging their contributions (Nembhard and Edmondson, 2006). It emphasizes a “people-oriented” approach, recognizing subordinates’ efforts with an equal attitude, and setting an example through personal effort (Liu et al., 2016). Carmeli et al. (2010) developed a three-dimensional scale for inclusive leadership, including openness, availability, and accessibility.

Transformational Leadership focuses on inspiring employees’ higher-level needs by changing followers’ attitudes, beliefs, and values, encouraging them to find meaning in their work (Bass, 1985). This leadership style encompasses four dimensions: charisma, inspiration, intellectual stimulation, and individualized consideration, which are measured by the Multifactor Leadership Questionnaire (MLQ) (Bass and Avolio, 1995). Subsequently, researchers proposed dimensions and questionnaires for transformational leadership in the Chinese context, consisting of moral example, vision inspiration, individualized consideration, and charisma (Li and Shi, 2005). Pearce et al. (2003) introduced the Four-Factor Leadership Theory, which categorizes leadership styles based on the degree of leader intervention into directive, transactional, transformational, and empowering leadership. Empowering Leadership involves sharing power with subordinates and enhancing their intrinsic motivation (Srivastava et al., 2006). Ahearne et al. (2005) categorized empowering leadership behaviours into enhancing the meaningfulness of work, promoting decision involvement, having confidence in subordinates, and providing autonomy, developing the Empowering Leadership Behaviour Scale. Similarly, Participative Leadership involves characteristics of involving employees in organizational decision-making. Leaders of this type negotiate with team members and share problem-solving solutions before making decisions (Bass, 1990). They empower teams to decide how to achieve goals and provide support for members’ self-management (Wageman, 2001). The widely used measurement tool for Participative Leadership is the six-item scale developed by Arnold et al. (2000).

In recent years, leadership styles based on moral ethics have also garnered attention from many researchers, including authentic leadership and servant leadership (Dinh et al., 2014). Authentic Leadership is a style characterized by behaviours consistent with one’s values, emphasizing genuine relationships with subordinates and colleagues and focusing on followers’ development (Gardner et al., 2005). Measurement of authentic leadership often utilizes scales developed by Walumbwa et al. (2008), which include dimensions such as self-awareness, relational transparency, information processing, and internalized morality. This scale has also been validated in the Chinese context (Wang et al., 2014). Eva et al. (2019) define Servant Leadership as a follower-centric approach prioritizing followers’ individual needs and interests and having a concern for others or even society as a whole. Van Dierendonck (2011) proposed six characteristics of servant leadership, including empowering and developing followers, humility, authenticity, interpersonal acceptance, providing direction, and taking responsibility. Some researchers include humble leadership within the realm of moral leadership (Lee et al., 2020). Humble Leadership is characterized by objective self-assessment, acknowledging one’s own shortcomings, appreciating others, and being more open to information (Owens and Hekman, 2012). Leaders of this type do not see themselves as superiors within the organization but actively integrate themselves among employees (Ou et al., 2014).

In addition to differential leadership, this study also includes other leadership styles with distinct characteristics of Chinese culture: authoritarian leadership, benevolent leadership, and moral leadership. Authoritarian Leadership is often seen as a destructive leadership style (Aryee et al., 2007). Leaders of this type make all decisions for the group and determine the steps to achieve goals (Lewin and Lippitt, 1938). Chinese researchers have pointed out that authoritarian leadership is a unique style among Chinese business leaders, characterized by dictatorship, belittling subordinates, and lecturing (Wang et al., 2010). Benevolent Leadership emphasizes leaders caring for subordinates like their own children, not only assisting subordinates in completing tasks in the workplace but also showing concern for their family and personal issues (Farh and Cheng, 2000). Moral Leadership refers to leaders possessing good moral qualities, emphasizing leading by example, separating public and private matters, and respecting the values of subordinates (Zhou and Long, 2007). Some researchers propose the concept of paternalistic leadership, considering authoritarian leadership, benevolent leadership, and moral leadership as three dimensions. They believe paternalistic leadership is “a leadership style that exhibits discipline, authority, paternal kindness, and moral integrity in an atmosphere of personal rule” (Farh and Cheng, 2000). Measurement is often conducted using the three-dimensional scale developed by Cheng et al. (2000), where each dimension corresponds to a specific leadership style. This scale has been proven to have good validity even in English contexts (Schaubroeck et al., 2017).

### Leadership and perceived insider status

Inducements and Contributions Theory (Stamper and Masterson, 2002) can provide theoretical foundations for studying the relationship between leadership styles and perceived insider status. Leaders control many “incentives” within the organization, such as training, promotion, and empowerment, they will provide these “incentives” to subordinates to imply that they have attained insider status (Wang et al., 2009). Therefore, the quality of the relationship between employees and leaders can significantly influence employees’ perception of insider status (Stamper and Masterson, 2002; Chen and Aryee, 2007). And according to a relational model of authority proposed by Tyler and Lind (1992), individuals determine their status in a group according to the way they are treated by authority figures. Based on this model, Lapalme et al. (2009) pointed out that perceptions of one’s relation with authorities are important indicators of one’s relation to the group.

Leader-Member Exchange (LMX) results in differential treatment of in-group and out-group members in terms of trust and respect (Graen and Uhl-Bien, 1995). Employees perceive themselves not only as “insiders” to their leader but also potentially as “insiders” to the organization, facilitating the development of perceived insider status (Shi et al., 2019). Chinese social relationships have the quality of self-centeredness, reflecting a hierarchical structure based on the closeness of relationships with others (Zhang et al., 2022). Differential leadership tends to classify subordinates as either “insiders” or “outsiders” and favour “insiders” in resource allocation. Inclusive leadership, characterized by openness and inclusiveness, makes employees feel recognized by the organization, enhancing their loyalty and sense of belonging (Wang et al., 2018). Likewise, perceived insider status reflects the extent to which employees feel recognized and valued in the organization. Participative leadership actively involves subordinates in decision-making and provides opportunities for them to take on greater responsibilities (Sauer, 2011). Inviting employees to participate in decision-making will provide incentives for employees, thereby enhancing their perceived insider status (Dou and Shen, 2021). Benevolent leadership provides subordinates with a safe and stable work environment, fostering trust between them and their leaders (Wu et al., 2012). Leaders’s act of benevolent helps establish a closer bond between individuals and the organization, enabling subordinates to perceive themselves as insiders (Shen et al., 2017).

Despite inducements-contributions theory and relational model of authority. There is another theory can provide explanations for employees’ perceived insider status. Social Identity Theory (Tajfel and Turner, 1986) posits that in order to reduce uncertainty and enhance self-esteem, individuals will categorize themselves and others into different groups to establish a self-concept (Ashforth and Mael, 1989). On one hand, leaders in organizations not only lead people, but they are also members of group. So, leader can become a prototypical group member, producing group influence and be accepted by employees (Van Knippenberg and Hogg, 2003). On the other hand, the way organizations treat individuals affects their attitudes and behaviours at work. When individuals feel appreciated and recognized by the organization, it signifies a higher status within the organization (Brown, 2000).

Transformational leadership inspires subordinates’ admiration and loyalty through its charisma, emphasizing the importance of the collective mission (Bass and Avolio, 1995). The leader’s individualized consideration makes recognition and encouragement as a “reward” for employees (Bass, 1990), and they can enhance employees’ empowerment (Avolio et al., 2004; Gumusluoglu and Ilsev, 2009). Empowering leadership shares information with employees, grants them rights, and gives them more participation in the organization (Yin et al., 2012). Moreover, empowering leadership, by providing more support and growth opportunities for subordinates, stimulates subordinates’ perceived insider status through the delegation of decision-making and autonomy (Zhao and Yan, 2016).

Authentic leadership serves as a role model for subordinates by reinforcing values of sincerity and integrity (Avolio and Gardner, 2005), fostering a trustworthy relationship with employees, and thereby allowing subordinates to perceive the leader’s reliability (Agote et al., 2015). The positive organizational environment created by authentic leadership meets employees’ needs, enhancing their perceived insider status (Zhao et al., 2021; Wang and Zhang, 2019). Servant leadership prioritizes individual needs, aims to cultivate and invest in followers’ interests and goals (Yeh et al., 2022; Opoku et al., 2019), and encourages them to develop a more favourable self-concept through social comparison processes (Opoku et al., 2019), helps establish strong relationships with employees, making them feel like partners in the organization (Eva et al., 2019). Humble leadership bravely admits mistakes to employees and maintains an inclusive attitude towards mistakes in work (Owens et al., 2013). Moreover, humble leadership discovers employees’ strengths, holds an open attitude towards learning from others, and listens to others’ advice and feedback (Owens and Hekman, 2012), thereby makes employees feel trusted and recognized by the leader (Su et al., 2017; Jia et al., 2023). Moral leadership, characterized by moral integrity, earns respect and emulation from subordinates, fostering harmonious leader-member relationships (Wu et al., 2012). Providing performance feedback to employees and granting them autonomy in their work enhances employees’ psychological empowerment (Li et al., 2012).

Authoritarian leadership is considered one of the destructive leadership styles, where leaders perceive themselves as the central source of information within the organization and believe they do not need to consult subordinates, viewing such consultation as a sign of incompetence and a threat to their authority (Cheng et al., 2000), which can constrain employees’ perception of themselves as part of the organizational collective (Schaubroeck et al., 2017). According to social identity theory, how individuals are treated by their superiors affects their perceived social status within the workgroup (Tyler and Blader, 2003). So authoritarian leadership, with its autocratic, demeaning, and authoritarian characteristics, makes it difficult for employees to perceive themselves as insiders, thus reducing their perceived insider status.

*Hypothesis 1:* Leader-member exchange, differential leadership, inclusive leadership, participative leadership, transformational leadership, empowering leadership, authentic leadership, servant leadership, humble leadership, benevolent leadership and moral leadership will positively relate to perceived insider status.

*Hypothesis 2:* Authoritarian leadership will negatively relate to perceived insider status.

Many studies have explored the relationship between leadership and perceived insider status, but how different leadership styles affect perceived insider status remains unclear. Which leadership style can have a greater impact? This study aims to comprehensively review the literature on leadership and perceived insider status, examining the differences in the impact of leadership styles on perceived insider status. Based on previous meta-analytical studies of leadership (Hoch et al., 2018; Xu and Li, 2019), this study uses transformational leadership as a benchmark, comparing it with other leadership styles pairwise to assess their contributions to perceived insider status. As mentioned above, leaders can enhance employees’ perceived insider status through several avenues: Firstly, leaders can improve relationships with subordinates, showing favouritism to make subordinates perceive themselves as having been bestowed with insider status in the organization (Jiang and Chang, 2010; Chen and Aryee, 2007). Secondly, perceived insider status refers to the feeling of organizational members about their own personal space and recognition within the organization (Masterson and Stamper, 2003). Leaders can enhance employees’ perceived insider status by granting them more autonomy to participate in organizational decision-making, thus experiencing more autonomy (Zhao and Yan, 2016; Avolio et al., 2004). Finally, leaders can serve as role models, conveying their exemplary power, and building trust relationships with employees, thereby enhancing employees’ sense of belonging in the organization (Eva et al., 2019).

When employees perceive that their contributions are valued and recognized by the organization, they are more likely to consider themselves insiders of the organization (Stamper and Masterson, 2002). Empowering leadership, by sharing power with subordinates and enhancing their intrinsic motivation (Srivastava et al., 2006), signals organizational identification to employees, thus exerting a positive impact on perceived insider status (Yin et al., 2012). Additionally, Wang et al. (2009) demonstrated in their study the positive effect of leader-member exchange on perceived insider status, attributing this effect to the support, empowerment, and trust that leaders provide to their in-group members—core traits of empowering leadership. In contrast, transformational leadership, prioritizing organizational goals over individual followers, may not always empower followers in certain situations, thus weakening followers’ autonomy (Sharma and Kirkman, 2015). And there is a study that have proposed the negative behavioural patterns of transformational leaders such as excessive self-importance and self-admiration and labelled these as authoritarian tendencies (Bass, 1990). This type of leadership will likely view the team as a vehicle to achieve their goal, even at the expense of other employees (Keeley, 1995). So, the dark side of transformational leadership may not be beneficial for the development of employee’s perceived insider status.

*Hypothesis 3:* Different leadership have varying explanatory power for perceived insider status, with empowering leadership contribute the greatest variance to perceived insider status and transformational leadership contribute the least variance to perceived insider status.

### Leadership and perceived insider status: moderation

#### Measurement tool

In studies related to leadership styles and perceived insider status, different measurement tools exist for the same leadership constructs, which may lead to differences in dimensions and content. Differences in measurement methods can affect the strength of relationships between variables; previous meta-analytical studies on LMX have shown that measurement methods play a significant moderating role (Gerstner and Day, 1997), although some research suggests otherwise (Dulebohn et al., 2012). Therefore, this study regards the measurement of leadership as a moderating variable and investigates whether it affects the relationship between leadership and perceived insider status.

#### Study design

The relationship between leadership styles and perceived insider status may be influenced by the research design, such as whether data are collected at the same time, which can be categorized into cross-sectional design and time-lagged study design. Some researchers have pointed out that cross-sectional research designs may affect significance tests and parameter estimation, and there may be a greater possibility of common method bias (Pitariu and Ployhart, 2010).

### Publication status

Generally, articles with significant results are more likely to be published, leading to an overestimation of the true effect sizes between variables in meta-analyses (Sterne et al., 2000). To avoid this bias, this study not only includes published journal articles but also incorporates theses to assess differences in results between published and unpublished articles.

### Demographic variables

Some studies have indicated that there are differences in the perception of the same leadership style between males and females (Lee and Park, 2021). The same leadership style can have different effects on employees of different genders (McCombs and Williams, 2021; Luo et al., 2022). Additionally, employees’ age may influence their understanding of leadership effectiveness, with older employees tending to have more positive evaluations of leadership compared to younger colleagues (Wang et al., 2019). Therefore, this meta-analysis also conducts moderator analyses on two demographic variables: gender and age.

## Method

### Literature search

A protocol for this work was registered on the Open Science Framework (OSF: https://osf.io/ph365) In order to identify published and unpublished samples that examined the relationship between leadership styles and perceived insider status. A thorough search was conducted. We searched Chinese databases including CNKI, Wanfang Data, and VIP Chinese Scientific Journals. English databases searched include ProQuest, Web of Science, and PubMed. The search terms combine “leadership” OR “leader” with “perceived insider status” OR “insider” OR “perceived insider identity.” The search includes publications up to December 2023.

According to the meta-analysis method and research requirements, the retrieved literature is screened based on the following criteria:

(a) Studies must be empirical research on the relationship between leadership and perceived insider status, excluding literature such as reviews, meta-analyses, and purely theoretical studies.
(b) Studies must utilize leadership scales and perceived insider status scales with good reliability and validity, and clearly report the sample size used in the study as well as the correlation data between variables.
(c) The study background must be within an organizational environment, with participants being employed staff. Studies conducted outside of workplace settings and involving participant groups such as students will be excluded.
(d) In cases of duplicate survey data, preference will be given to published journal articles.

**Figure 1:**
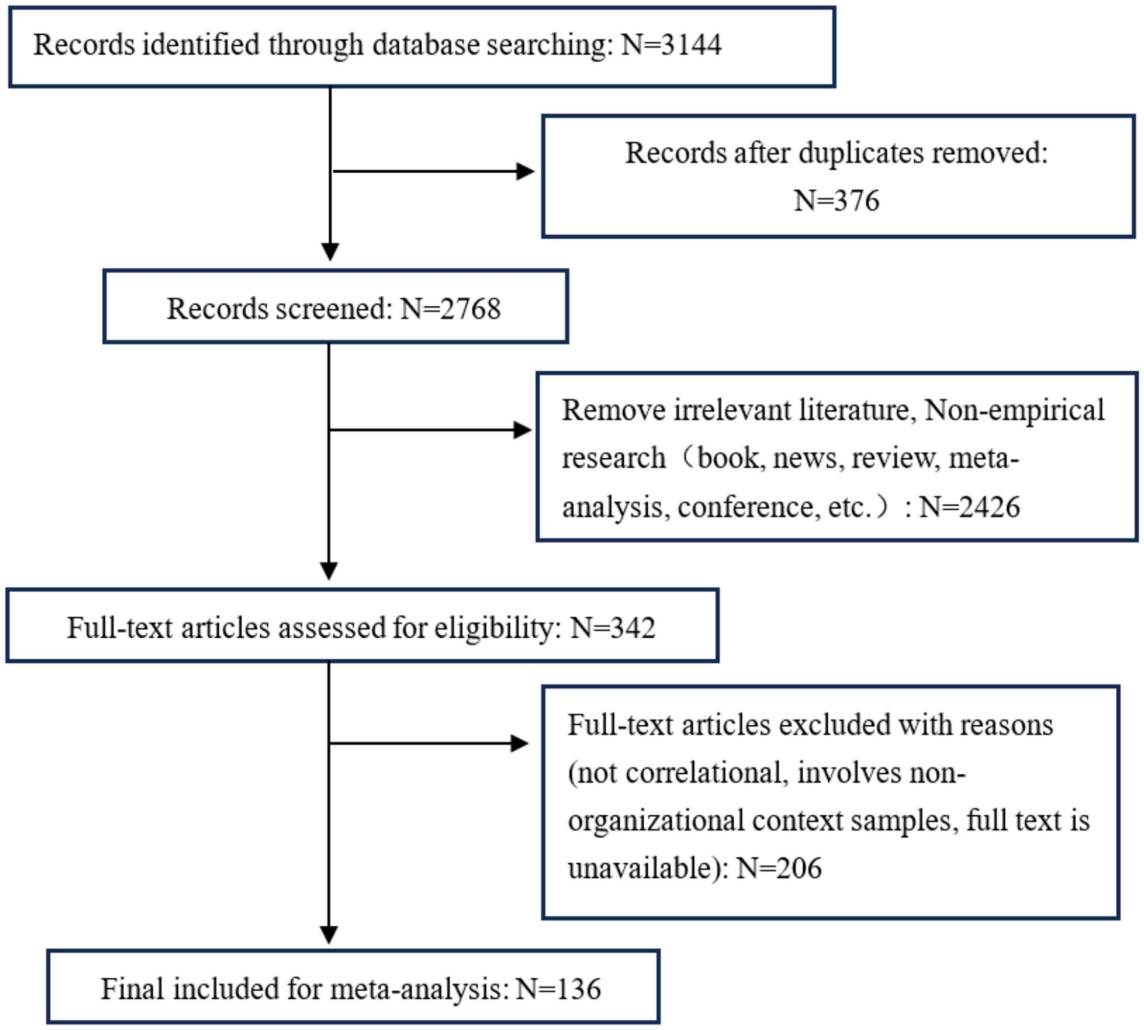
Flow diagram illustrating the process of our review, screening, and article selections.

The literature screening was conducted according to the above standards, and the specific screening process is illustrated in Figure 1. In total, 137 articles meeting the criteria were collected, including 118 in Chinese and 20 in English, comprising 70 journal articles and 67 theses. This resulted in 151 independent samples, covering 45,228 participants.

### Coding procedures

Coding of literature included in the meta-analysis (see Supplementary Table S2) includes information such as authors, publication year, sample size, correlation coefficient, reliability, leadership type, leadership measurement tool, study design, and publication status. Each independent sample needs to be coded separately. If a study does not report an overall correlation but only reports the correlation between each dimension of leadership and perceived insider status, the mean will be calculated to represent the overall correlation (Hunter and Schmidt, 2004; Xue et al., 2022).

### Meta-analytic procedures

This study utilizes CMA3.0 for conducting meta-analysis tests. The correlation coefficient (r) is used as the effect size, and each effect size are corrected using reliability coefficients. For studies with missing reliability information, we used the mean of reliabilities of the studies that reported reliability information, and ultimately, the true correlation coefficient (*ρ*) is calculated (Hunter and Schmidt, 2004). We also calculated corrected 95% confidence intervals to determine the statistical significance of effect sizes. If the confidence intervals do not include zero, effect sizes are considered to be statistically significant.

For relative weight analysis, we combined effect sizes from the present study and prior meta-analytic to construct a meta-analytic correlation matrix. Relative importance analysis is carried out using RWA Web to compare the impact of various leadership styles on perceived insider status (Tonidandel and LeBreton, 2015; Xue et al., 2022).

We ran random-effects model to examined moderators, subgroup analysis and meta-regression were used separately to determine whether there exists significant difference between continuous or categorical moderators. The categorical moderators we included in subgroup analysis were leadership measurement tools, study design (whether leadership styles and perceived insider status are measured at the same time) and publication status (Whether the article has been published or not). These were all dummy coded. The continuous moderators we included were gender and age.

## Results

### Homogeneity test

This study conducted a homogeneity test on the included effect sizes to determine whether the effect sizes are homogeneous, thus deciding whether subsequent data analysis should be based on a fixed-effect model or a random-effects model. The results in Table 1 indicate that the Q statistics for each leadership style reach significance (*ps*< 0.01), suggesting heterogeneity among the effect sizes. An *I*^2^ value exceeding 75% indicates a high degree of heterogeneity, while *τ^2^* indicates the extent to which variability between studies can be used to calculate weights. Therefore, given the heterogeneity in effect sizes between various leadership styles and perceived insider status, a random-effects model was selected for the study.

**Table 1:** The homogeneity tests.

Variable	k	*N*	Homogeneity			Tau-squared
			Q	df (Q)	I^2^	
Leader-member exchange	35	11,148	988.77^***^	34	96.56	0.09
Differential leadership	20	8,014	2321.80^***^	19	99.18	0.31
Inclusive leadership	17	5,495	655.91^***^	16	97.56	0.13
Participative leadership	4	1,142	24.03^***^	3	87.52	0.03
Transformational leadership	6	2,110	126.48^***^	5	96.05	0.07
Empowering leadership	16	4,955	349.31^***^	15	95.71	0.07
Authentic leadership	6	2,226	65.33^***^	5	92.35	0.03
Servant leadership	10	2,958	145.80^***^	9	93.83	0.05
Humble leadership	13	4,422	354.69^***^	12	96.62	0.09
Authoritarian leadership	11	3,472	184.37^***^	10	94.58	0.06
Benevolent leadership	9	3,081	20.31^**^	8	60.61	0.01
Moral leadership	4	1,633	12.17^**^	3	75.34	0.01

*p < 0.05, **p < 0.01, ***p < 0.001. The asterisks attached to the Q statistic represent the significance level for the test of homogeneity.

### Publication bias

Publication bias refers to the bias caused by the insufficient representation of the total research population in the published studies. This bias may result from systematic sampling or selective reporting in individual studies, as well as from researchers’ omission of important literature during the literature search process. In this study, three methods, including Egger’s regression coefficient test, Begg’s rank correlation test, and the trim and fill method, were employed to assess publication bias in the literature included on leadership and perceived insider status. The results are presented in Table 2. According to the results of the trim and fill method, all effects meet the 5 k + 10 criterion. The *p*-values of Egger’s regression coefficient and Begg’s rank correlation tests for each leadership are not significant (*p*s > 0.05). Furthermore, to assess potential publication bias, we employed both Duval and Tweedie’s Trim-and-Fill method and the PET-PEESE framework, the results are presented in Table 3. The Trim-and-Fill method indicated that even after adjusting for potentially missing studies, the overall effect remains significant, suggesting no serious publication bias issues in this meta-analysis. Consequently, we report the PET intercept as the bias-adjusted estimate, which remained statistically significant. The convergence of these methods suggests our findings are robust against publication bias.

**Table 2:** Publication bias.

Variable	Begg and Mazumdar rank correlation		k	Fail Safe k	Egg’s regression				
	Z	*p*			intercept	SE	t	df	*p*
Leader-member exchange	1.73	0.08	34	4,445	0.96	4.14	0.23	33	0.82
Differential leadership	1.33	0.18	20	3,677	−17.11	11.46	1.49	18	0.15
Inclusive leadership	0.29	0.77	17	3,048	−7.14	10.45	0.68	15	0.50
Participative leadership	0.00	1.00	4	198	7.41	16.96	0.44	2	0.70
Transformational leadership	0.35	0.71	6	647	−10.85	21.76	0.50	4	0.64
Empowering leadership	1.13	0.26	16	8,639	−11.24	8.82	1.27	14	0.22
Authentic leadership	0.45	0.65	10	3,208	9.42	13.66	0.69	8	0.51
Servant leadership	2.44	0.01*	6	1,215	−35.74	21.94	1.63	4	0.17
Humble leadership	0.48	0.95	13	5,393	0.66	7.71	0.09	11	0.93
Authoritarian leadership	0.31	0.76	11	546	7.43	7.54	0.99	9	0.35
Benevolent leadership	0.52	0.60	9	1908	−1.31	3.47	0.38	7	0.72
Moral leadership	0.34	0.73	4	672	−6.88	5.17	1.33	2	0.31

**Table 3:** Adjusted effect sizes under different publication bias methods.

Variable	Analysis model	ρ	95% CI
Leader-member exchange	Trim-and-fill adjustment	0.36	[0.30, 0.42]
	PET-PEESE framework	0.33	[0.11, 0.51]
Differential leadership	Trim-and-fill adjustment	0.40	[0.27, 0.53]
	PET-PEESE framework	0.31	[0.01, 0.56]
Inclusive leadership	Trim-and-fill adjustment	0.50	[0.46, 0.62]
	PET-PEESE framework	0.48	[0.21, 0.68]
Participative leadership	Trim-and-fill adjustment	0.32	[0.19, 0.44]
	PET-PEESE framework	0.25	[−0.33, 0.69]
Transformational leadership	Trim-and-fill adjustment	0.36	[0.20, 0.50]
	PET-PEESE framework	0.31	[−0.15, 0.66]
Empowering leadership	Trim-and-fill adjustment	0.49	[0.40, 0.57]
	PET-PEESE framework	0.38	[0.02 0.65]
Authentic leadership	Trim-and-fill adjustment	0.48	[0.43, 0.53]
	PET-PEESE framework	0.48	[0.32, 0.62]
Servant leadership	Trim-and-fill adjustment	0.52	[0.43, 0.61]
	PET-PEESE framework	0.46	[0.25, 0.62]
Humble leadership	Trim-and-Fill adjustment	0.48	[0.38, 0.57]
	PET-PEESE framework	0.41	[0.16, 0.60]
Authoritarian leadership	Trim-and-Fill adjustment	0.48	[−0.29, −0.08]
	PET-PEESE framework	−0.22	[−0.38, −0.05]
Benevolent leadership	Trim-and-Fill adjustment	0.41	[0.38, 0.45]
	PET-PEESE framework	0.37	[0.25, 0.49]
Moral leadership	Trim-and-Fill adjustment	0.49	[0.42, 0.53]
	PET-PEESE framework	0.45	[0.31, 0.57]

### Meta-analysis

**Figure 2:**
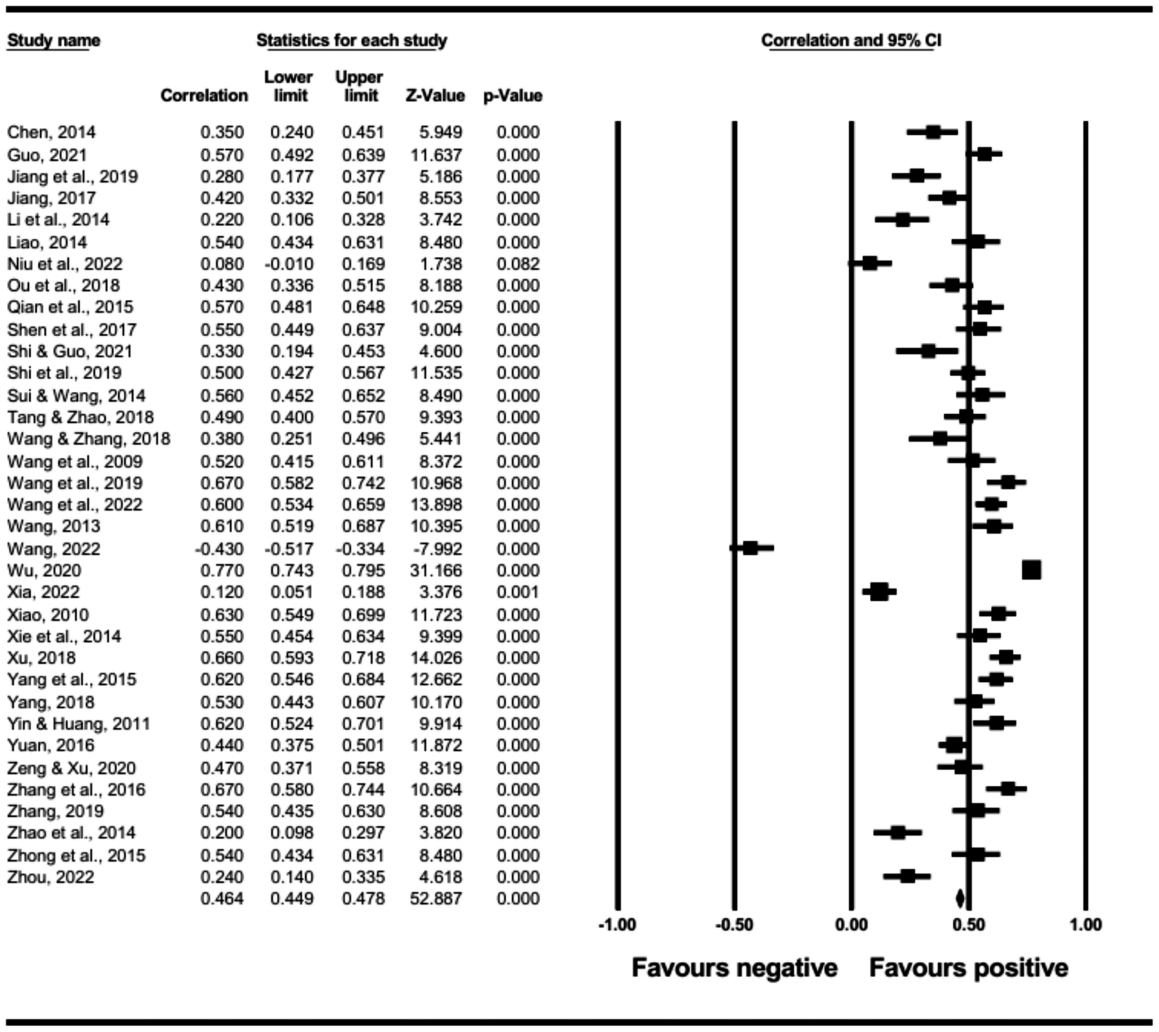
The association between leader-member exchange and perceived insider status.

**Figure 3:**
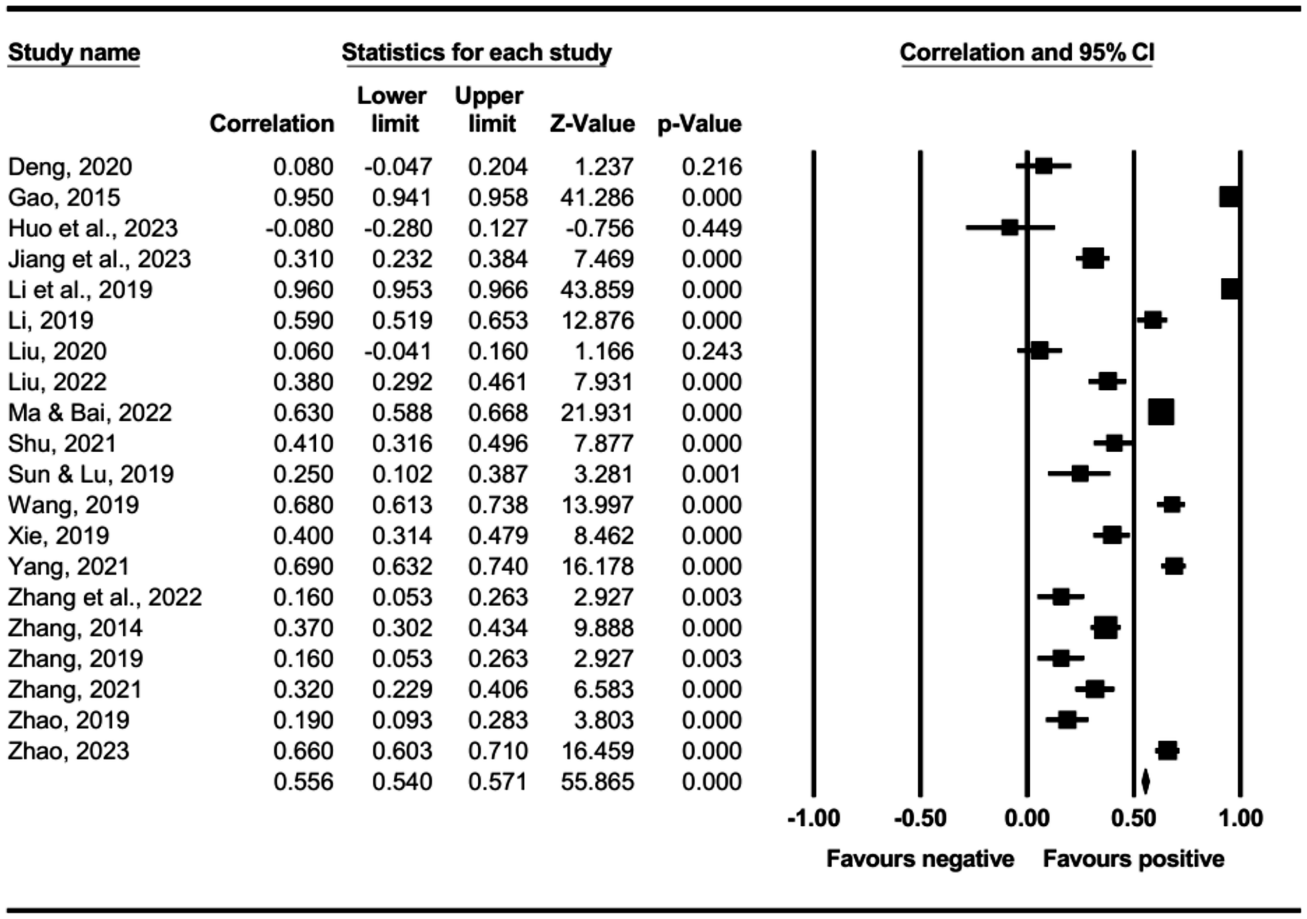
The association between differential leadership and perceived insider status.

**Figure 4:**
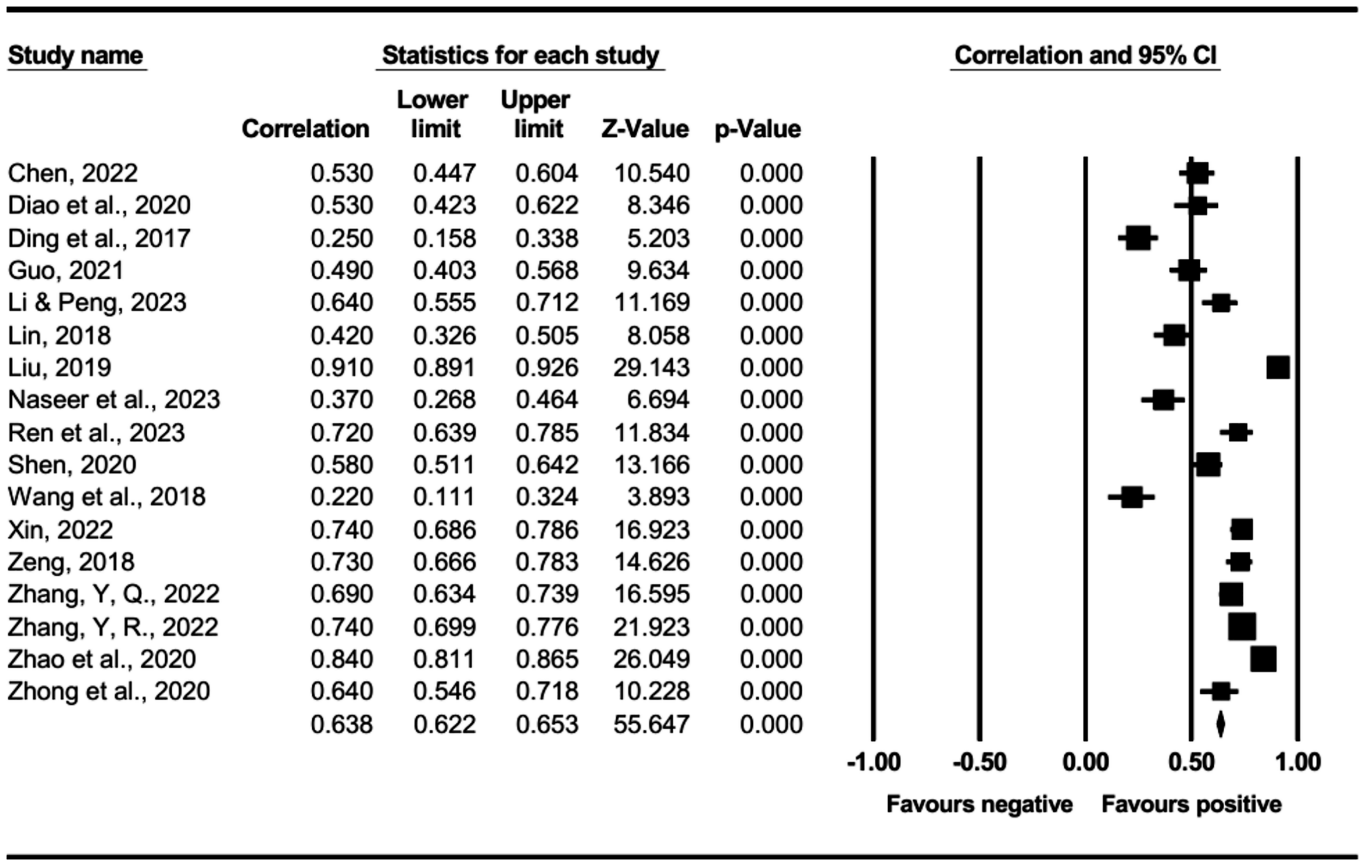
The association between inclusive leadership and perceived insider status.

**Figure 5:**
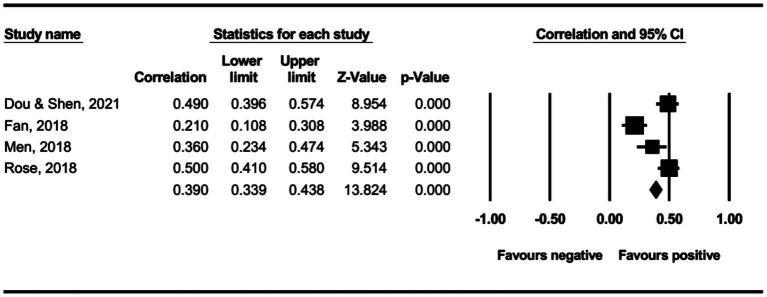
The association between participative leadership and perceived insider status.

**Figure 6:**
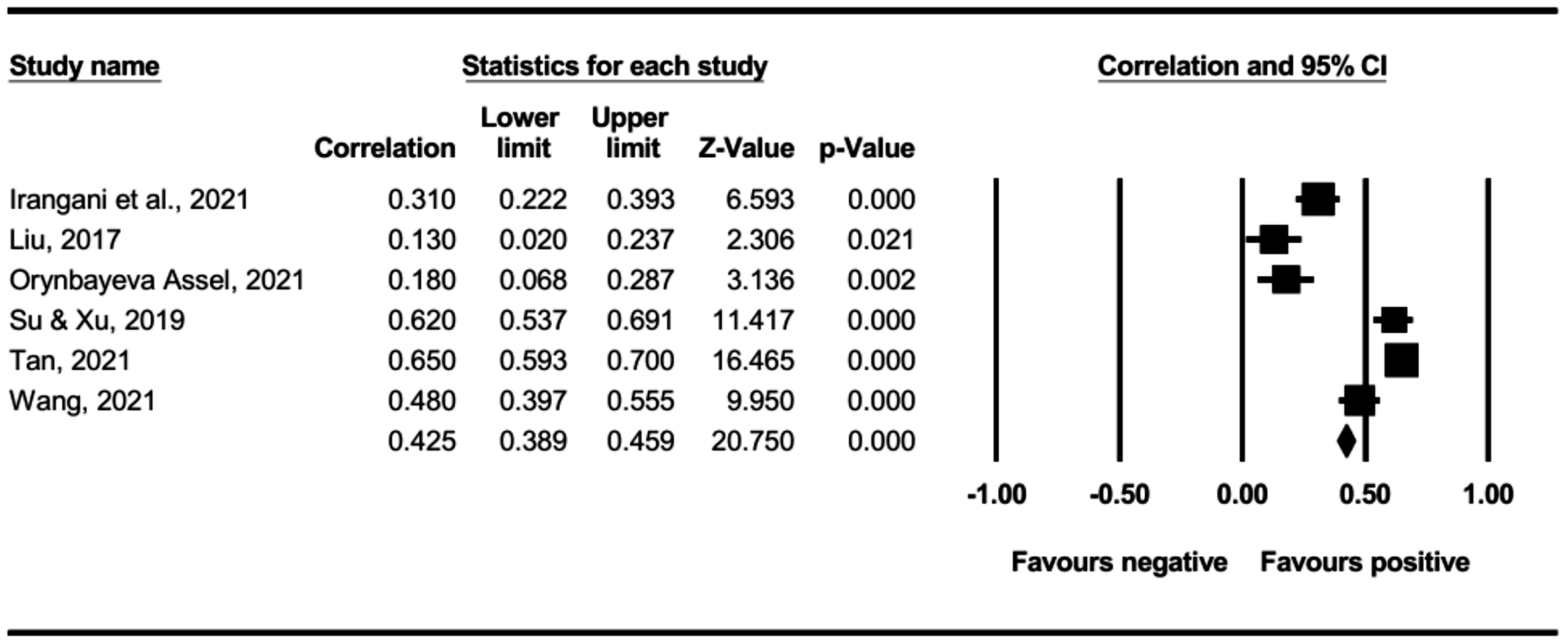
The association between transformational leadership and perceived insider status.

**Figure 7:**
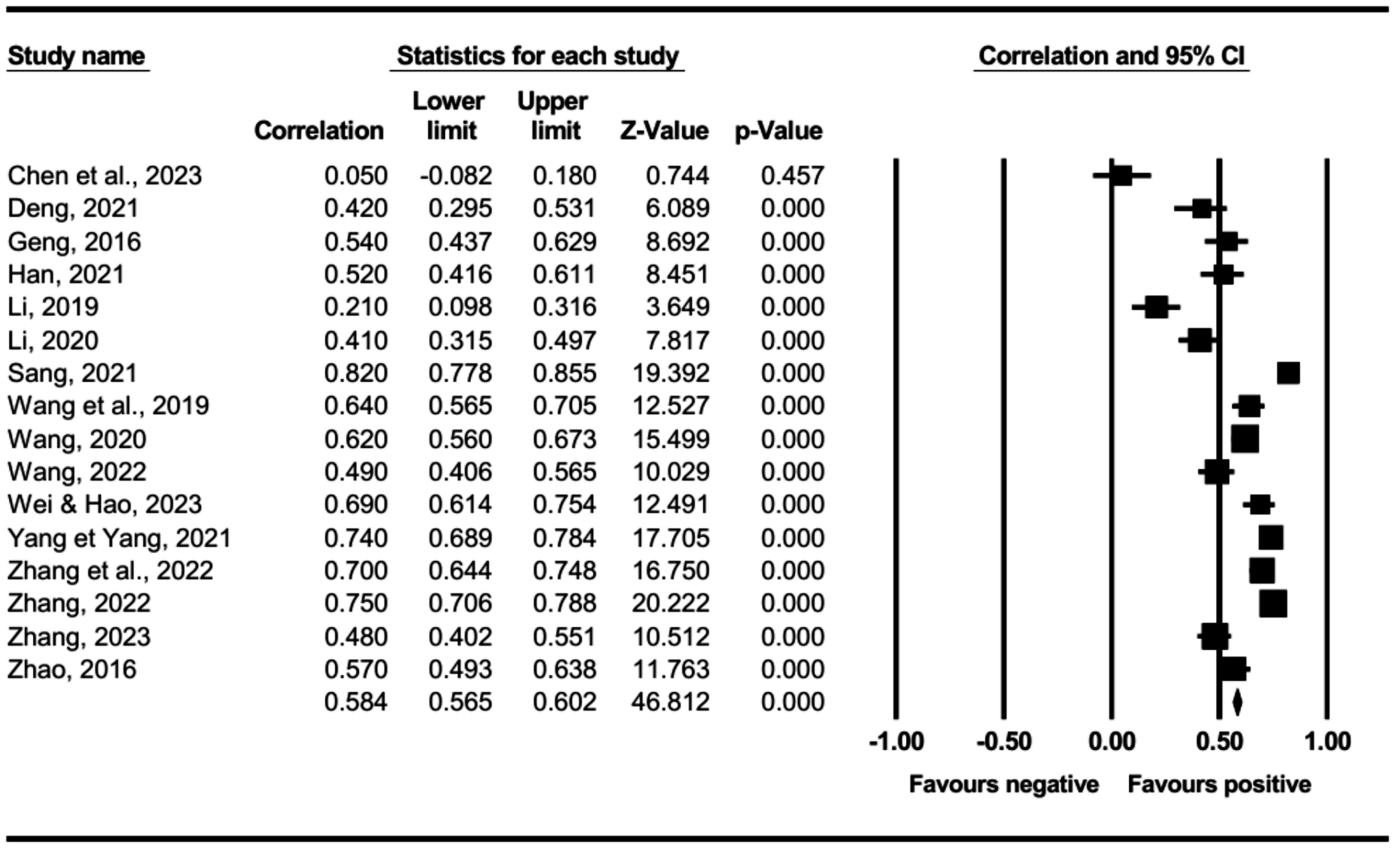
The association between empowering leadership and perceived insider status.

**Figure 8:**
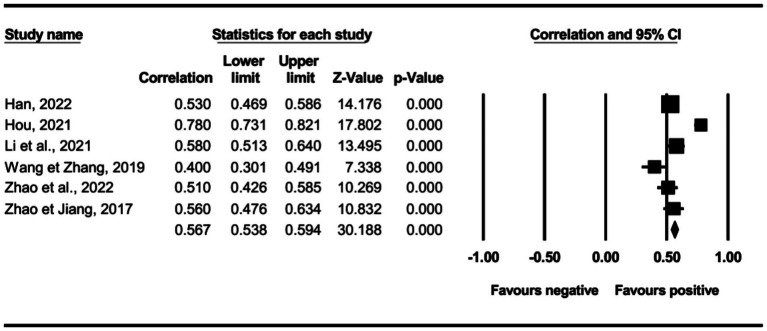
The association between authentic leadership and perceived insider status.

**Figure 9:**
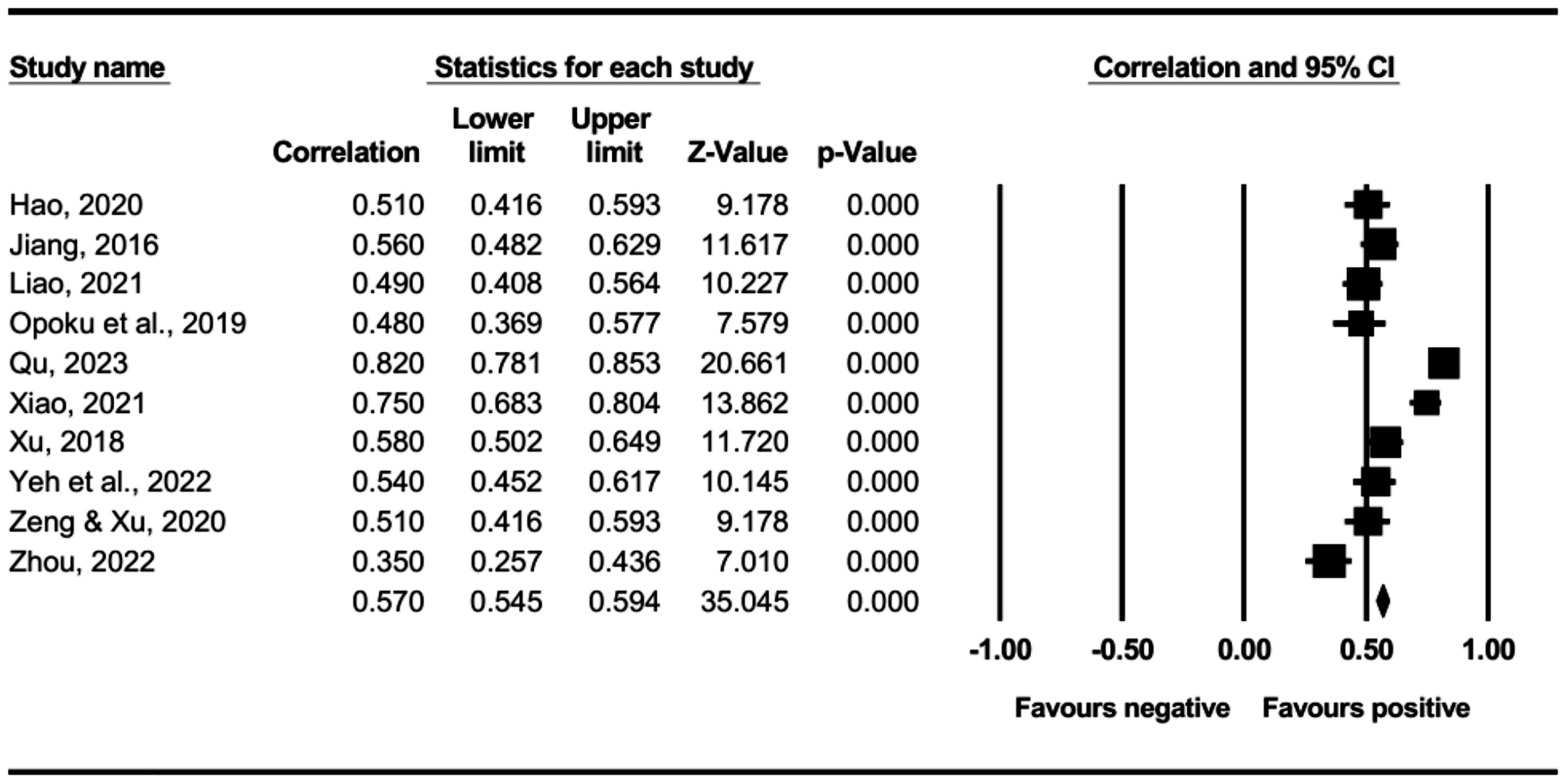
The association between servant leadership and perceived insider status.

**Figure 10:**
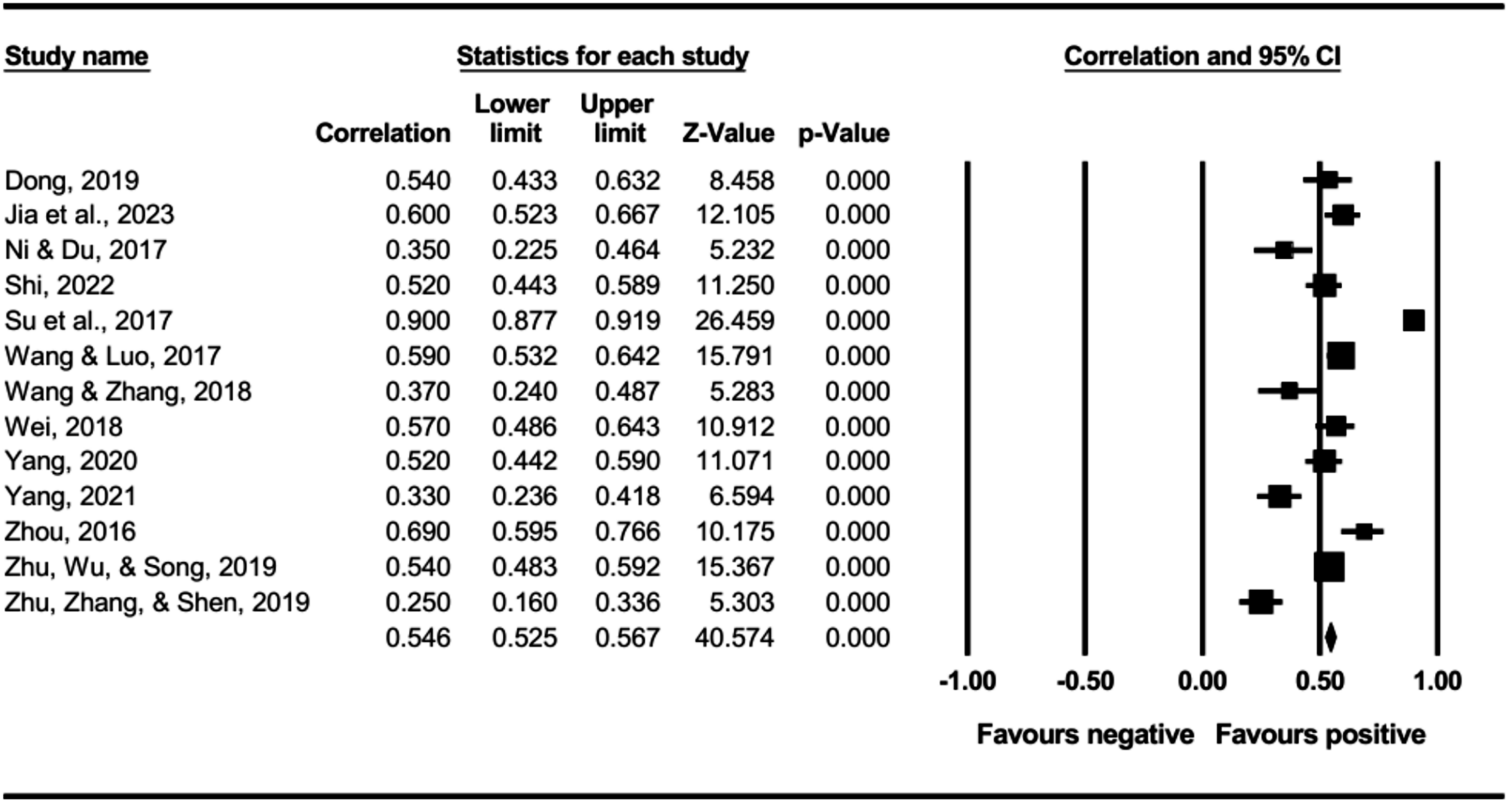
The association between humble leadership and perceived insider status.

**Figure 11:**
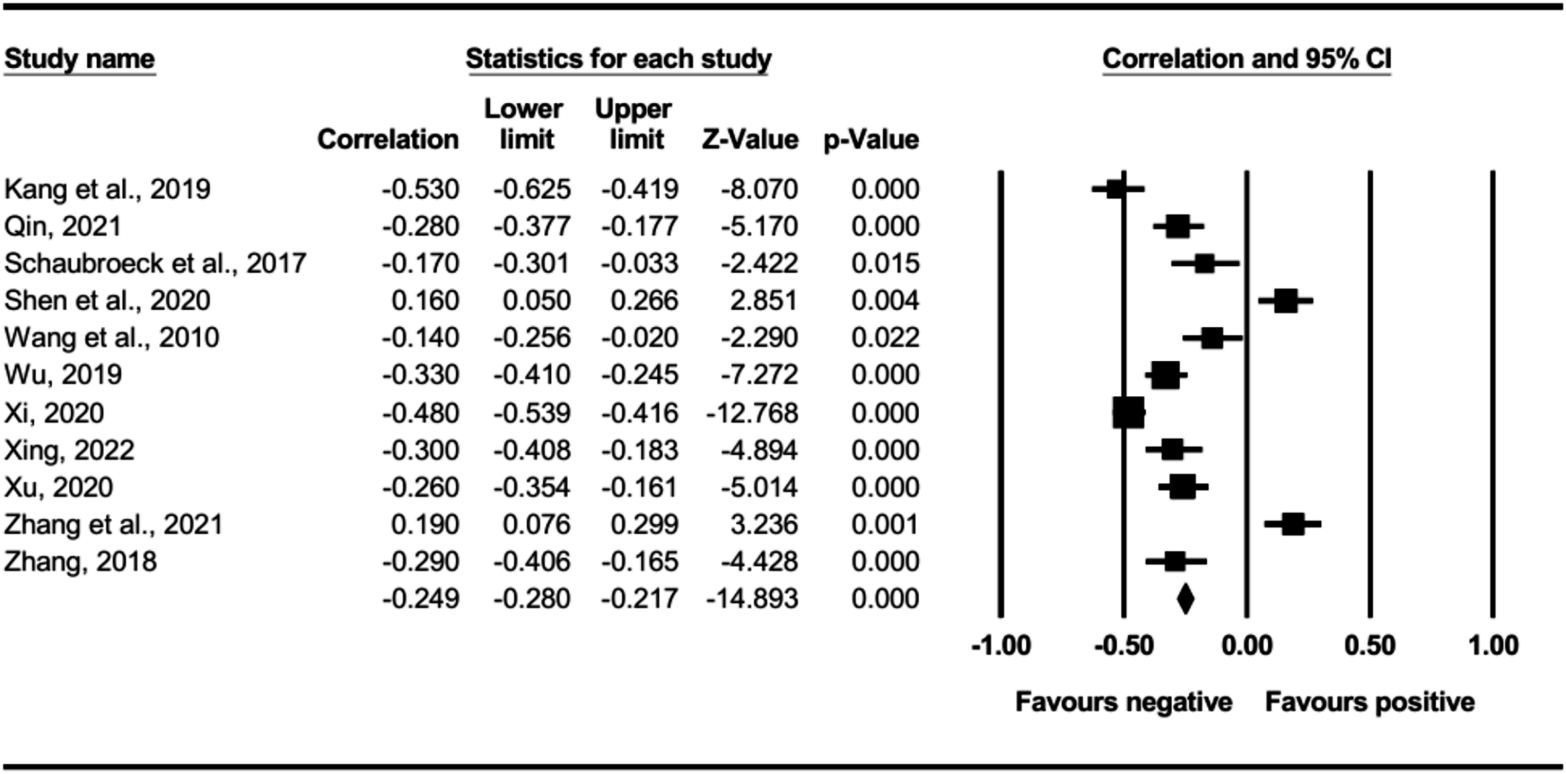
The association between authoritarian leadership and perceived insider status.

**Figure 12:**
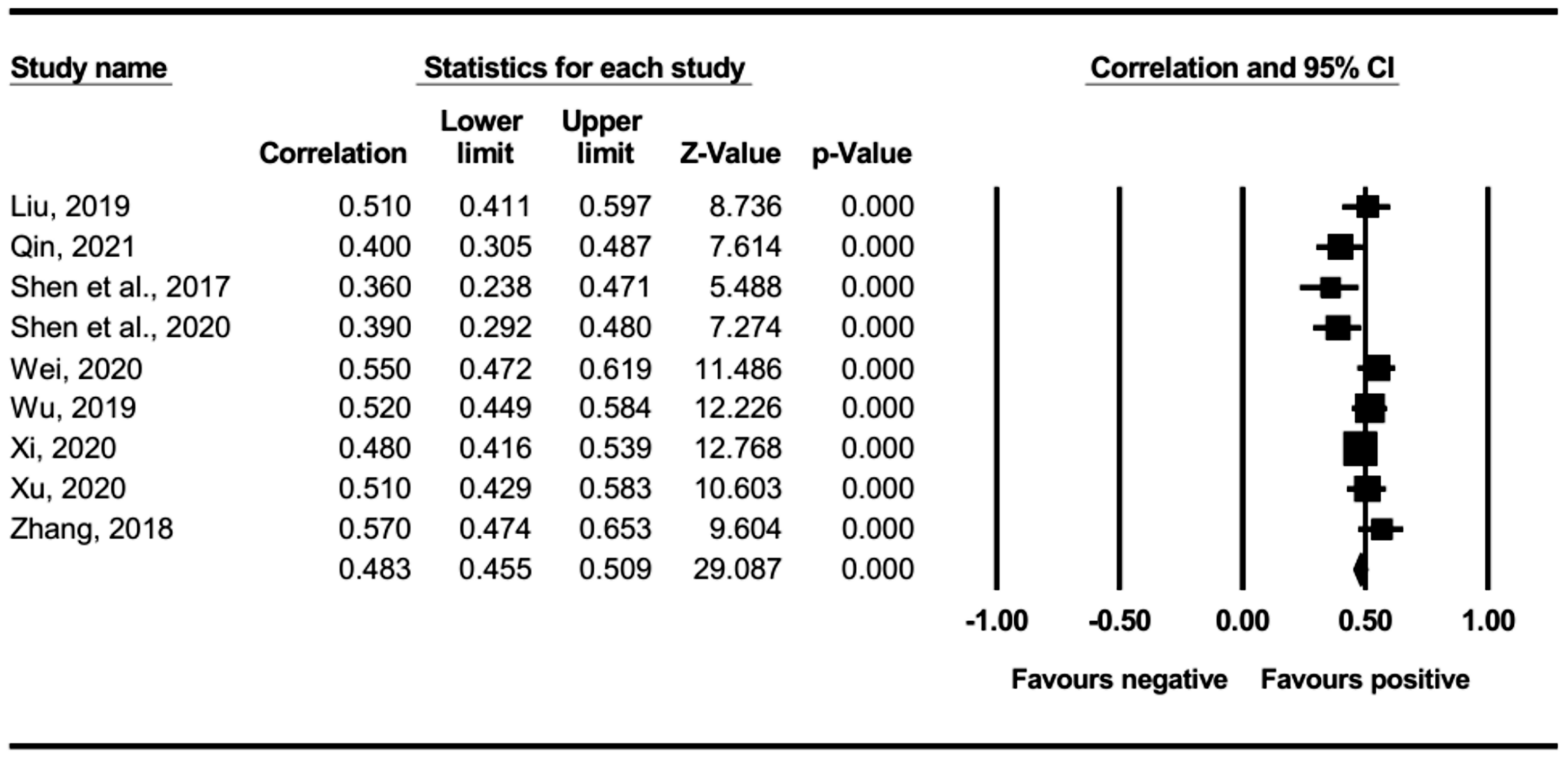
The association between benevolent leadership and perceived insider status.

**Figure 13:**
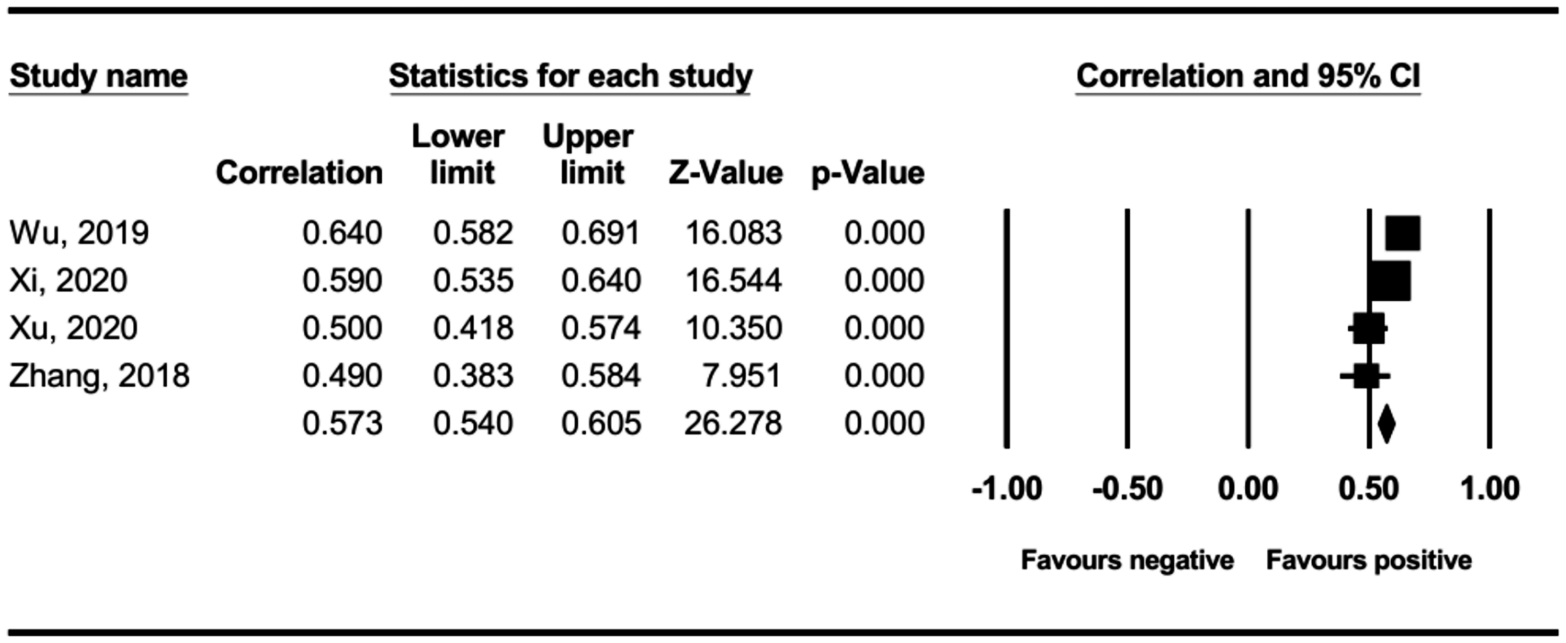
The association between moral leadership and perceived insider status.

The main effect test results of each leadership style on perceived insider status are presented in Table 3, and forest plot analyses are presented in Figures 2–13. The results indicate significant positive correlations (*p*s< 0.001) between leadership styles, including Leader-Member Exchange (*ρ* = 0.47), Differential Leadership (*ρ* = 0.49), Inclusive Leadership (*ρ* = 0.63), Participative Leadership (*ρ* = 0.40), Transformational Leadership (*ρ* = 0.42), Empowering Leadership (*ρ* = 0.57), Authentic Leadership (*ρ* = 0.57), Servant Leadership (*ρ* = 0.58), Humble Leadership (*ρ* = 0.55), Benevolent Leadership (*ρ* = 0.48), Moral Leadership (*ρ* = 0.56), and perceived insider status. Additionally, Authoritarian Leadership (*ρ* = −0.23) shows a significant negative correlation (*p* < 0.05) with perceived insider status. These results support Hypotheses 1 and Hypotheses 2.

### Relative weight analysis

To determine the differential effects of leadership styles on explaining perceived insider status, this study utilized the relevant structures of leadership from previous research to form a correlation matrix (Table 4). The results of relative weight analysis are presented in Table 5, indicating differences in the explanatory power of different leadership styles for perceived insider status. Except for missing correlations between differential leadership, participative leadership and transformational leadership, all other correlations are accounted for. Compared to transformational leadership, leader-member exchange, inclusive leadership, empowering leadership, authentic leadership, servant leadership, humble leadership, benevolent Leadership, and moral leadership demonstrate stronger explanatory power for perceived insider status. Among these, inclusive leadership has the most significant impact on perceived insider status. Thus, Hypotheses 3 is partially supported (see Table 6).

**Table 4:** Bivariate relationships between leadership and perceived insider status.

Variable	k	*n*	r	ρ	95% CI	z-value	*p*
Leader-member exchange	35	11,198	0.40	0.47	[0.39, 0.55]	9.93	<0.001
Differential leadership	20	8,014	0.41	0.49	[0.29, 0.66]	4.34	<0.001
Inclusive leadership	17	5,495	0.54	0.63	[0.51, 0.72]	8.47	<0.001
Participative leadership	4	1,142	0.32	0.40	[0.25, 0.53]	4.93	<0.001
Transformational leadership	6	2,110	0.36	0.42	[0.22, 0.58]	4.02	<0.001
Empowering leadership	16	4,955	0.49	0.57	[0.47, 0.65]	9.34	<0.001
Authentic leadership	6	2,226	0.49	0.57	[0.46, 0.67]	8.40	<0.001
Servant leadership	10	2,958	0.52	0.58	[0.47, 0.67]	8.81	<0.001
Humble leadership	13	4,422	0.48	0.55	[0.43, 0.65]	7.46	<0.001
Authoritarian leadership	11	3,472	−0.19	−0.23	[−0.36, −0.09]	−3.17	<0.05
Benevolent leadership	9	3,081	0.41	0.48	[0.44, 0.52]	17.87	<0.001
Moral leadership	4	1,633	0.48	0.56	[0.49, 0.63]	12.40	<0.001

**Table 5:** Meta-analytic estimates of intercorrelation among study variables.

Variable	PIS	LMX	DL	IL	PL	TL	EL	AL	SL	HL	AUL	BL	ML
PIS	1	0.47	0.49	0.63	0.40	0.42	0.57	0.57	0.58	0.55	−0.23	0.48	0.56
K (N)	-	35 (11198)	20 (8014)	17 (5495)	4 (1142)	6 (2110)	16 (4955)	6 (2226)	10 (2958)	13 (4422)	11 (3472)	9 (3081)	4 (1633)
TL	-	0.71a	-	0.59e	-	-	0.73c	0.75a	0.52a	0.8d	−0.29b	0.71b	0.74b
K (N)	-	20 (4591)	-	2 (315)	-	-	3 (469)	10 (2397)	5 (774)	3 (497)	12 (3829)	10 (3671)	11 (3785)

PIS = Perceived insider status; LMX = Leader-member exchange; DL = Differential leadership; IL = Inclusive leadership; PL = Participative leadership; TL = Transformational leadership; EL = Empowering leadership; AL = Authentic leadership; SL = Servant leadership; HL = Humble leadership; AUL = Authoritarian leadership; BL = Benevolent leadership; ML = Moral leadership. Unless stated, meta-analytic correlations were calculated by authors. (a) Hoch et al. (2018); (b) Hiller et al. (2019); (c) Xu and Li (2019); (d) Luo et al. (2022); (e) Lin et al. (2021).

**Table 6:** Relative weight analysis.

Variable	Raw relative weights	Rescaled relative weights %	R-square
TL	0.10	40.57	0.24
LMX	0.14	59.43	
TL	0.09	22.47	0.40
IL	0.31	77.53	
TL	0.09	27.15	0.32
EL	0.24	72.85	
TL	0.09	27.16	0.33
AL	0.24	72.84	
TL	0.10	27.50	0.36
SL	0.26	72.50	
TL	0.09	29.23	0.30
HL	0.16	70.77	
TL	0.09	38.89	0.24
BL	0.15	61.11	
TL	0.09	28.13	0.31
ML	0.23	71.87	

TL = Transformational leadership; LMX = Leader-member exchange; IL = Inclusive leadership; EL = Empowering leadership; AL = Authentic leadership; SL = Servant leadership; HL = Humble leadership; BL = Benevolent leadership; ML = Moral leadership.

### Moderator analyses

This study focuses on several potential moderating variables, and the moderation effects are tested using a random-effects model. For categorical moderating variables, subgroup analysis is then conducted to test the moderation effects, excluding some subgroups with effect sizes smaller than 3. For continuous moderating variables, the mean values are calculated, and meta-regression analysis is employed to test the moderation effects. Some variables with effect sizes smaller than 10 are not analysed. The results of moderation effects are presented in Supplementary Tables S3, S4, indicating that the moderation effects of study design, leadership measurement tools, publication status, gender and age are all not significant.

## Discussion

At present, numerous studies have examined the relationship between leadership and perceived insider status, yet there remains a lack of systematic and quantitative reviews of this relationship. This paper utilizes meta-analysis techniques to examine the impact of leadership styles on perceived insider status and the role of potential moderating variables. The results indicate significant positive correlations between leader-member exchange, differential leadership, inclusive leadership, participative leadership, transformational leadership, empowering leadership, authentic leadership, servant leadership, humble leadership, benevolent leadership, moral leadership and perceived insider status. Additionally, authoritarian leadership shows a significant negative correlation with perceived insider status. Furthermore, this study integrates previous meta-analyses related to leadership styles to form a correlation matrix between variables and conducts a relative weight analysis to explore the degree to which different leadership styles influence perceived insider status. These results provide a comprehensive understanding of the relationship between leadership styles and perceived insider status and clarify discrepancies in previous research.

In organizations, leadership is one of the key factors influencing employee behaviour. Business leaders tend to classify subordinates who have high-quality leadership exchanges as insiders, providing opportunities such as positions, salaries, and training (Wang et al., 2009), and treat insiders with favouritism (Cheng, 1995), thereby enhancing subordinates’ perceived insider status. In addition to relational leadership, other leadership traits such as encouraging employees to propose new ideas, maintaining an open attitude towards different viewpoints, and providing support to employees when they encounter difficulties can enhance members’ sense of belonging in the organization (Naseer et al., 2023; Dou and Shen, 2021), thereby making employees feel like “insiders” in the organization. Likewise, the moral character of the leader can also improve employee’s perceived insider status. Leader sets an example through their own morals and values, which allows subordinates to experience positive emotions establishing a role model image among subordinates (Avolio and Gardner, 2005). They prioritize followers’ needs, creating an atmosphere of encouragement and care (Yeh et al., 2022) and are willing to acknowledge their own mistakes, recognize employees’ contributions, and maintain an open attitude. Employees can share their genuine thoughts and suggestions with leaders, feeling that the organization sees them as internal members (Jia et al., 2023). On the contrary, there is one leadership style negatively relates to perceived insider status in our meta-analysis, that is authoritarian leadership. It is a leadership style originated from high power distance societies such as China, and in our study, the samples of authoritarian leadership were all from China. This kind of leader emphasizes personal dignity and tends to belittle subordinates, which hinders the formation of high-quality member relationships with employees, making it difficult for employees to perceive themselves as internal to the organization.

The results of relative weight analysis indicate that different leadership styles have varying effects on perceived insider status. Inclusive leadership, empowering leadership, authentic leadership, servant leadership, humble leadership, leader-member exchange, and transformational leadership have diminishing explanatory power on perceived insider status. In previous studies, transformational leadership is often associated with positive organization outcomes (Bass, 1985; Resick et al., 2009), but in this meta-analysis, transformational leadership, compared to other leadership styles, exhibits the lowest explanatory power on perceived insider status. This might be because, unlike some leadership styles that focus on follower traits, transformational leadership is more oriented towards organizational goals (Hoch et al., 2018). The charisma, as core of this leadership, is possible to evolve into boastfulness, self-center and addicted to power and superiority (De Villiers, 2014). Additionally, in certain contexts, transformational leadership may not delegate authority to followers; they may demonstrate transformational behaviours without actually transferring control or rights to followers (Sharma and Kirkman, 2015). Xue et al. (2022) also demonstrated in their meta-analysis that empowering leadership and servant leadership explain more intrinsic motivation differences compared to transformational leadership. In Hoch et al.'s (2018) meta-analysis, it was found that compared to transformational leadership, authentic leadership, ethical leadership, and servant leadership provide greater explanatory power in organizational commitment, affective commitment, and trust in leadership, all of which are important antecedents of perceived insider status (Xia et al., 2022).

Based on the meta-analytic findings, inclusive leadership emerged as the most dominant leadership style in fostering perceived insider status, demonstrating a stronger explanatory power than other styles, including empowering leadership. The potency of inclusive leadership can be attributed to its direct alignment with the core psychological dimensions of PIS.

While empowering leadership enables delegation of power and provides learning opportunities for employees (Lee et al., 2017), allows employees to have more participation in the organization, fostering a sense of trust from both leadership and the organization, thereby stimulating employees’ perceived insider status. However, inclusive leadership operates on a more fundamental level. Some researchers define inclusive leadership as “the extent to which employees in the workplace experience treatment that satisfies their needs for belongingness and uniqueness, thereby perceiving themselves as respected members of the work group” (Randel et al., 2018). This leadership enhances subordinates’ job identification and psychological empowerment, facilitating positive interaction between leaders and subordinates. Additionally, it promotes employees’ sense of belonging within the organization and clarifies their uniqueness. Employees can feel recognized by the organization, and perceived insider status reflects the degree to which employees perceive themselves as recognized and valued within the organization. Consequently, employees under inclusive leadership are more likely to perceive themselves as both integral and indispensable members of the organization. This profound sense of being recognized and valued for who they are explains why inclusive leadership was found to be the most dominant predictor of perceived insider status in this study.

Moreover, compared to other leadership styles, transformational leadership can leverage its charisma, while empowering leadership can facilitate members’ control over decision-making and goals. However, neither of these approaches enhances members’ sense of belonging in an inclusive manner. Leader-member exchange focuses on resource exchanges between dyads, whereas inclusive leadership places greater emphasis on members within the workgroup, aiding employees in feeling a sense of belonging and valuing differences within the workgroup (Galvin et al., 2010; Randel et al., 2018). Therefore, inclusive leadership may have a greater impact on perceived insider status.

The meta-analysis results indicate that study design, measurement tools of leadership, publication status, gender, and age do not have significant moderation effects on the relationship between leadership styles and perceived insider status. Previous meta-analyses have shown that using homogeneous data may lead to increased correlation between samples (Lee et al., 2020), such as employing self-report measures or collecting questionnaires at the same time. Even though no significant moderation effects were found between data collected at single time points and multiple time points in this study, caution should still be exercised in future research to avoid common method bias, which could inflate the correlation between variables. Measurement tools of leadership have also been found to exhibit significant differences in previous meta-analyses. In the study by Lin et al. (2022), the measurement tool of transformational leadership showed significant moderation effects. Therefore, the influence of measurement tools should continue to be considered in future research. Reviewing literature in journals reveals a higher proportion of studies with statistically significant results. Results from O’Boyle et al. (2017) also indicate that the ratio of studies supporting hypotheses to those not supporting hypotheses doubled in published articles compared to those in dissertations. Although the moderation variables examined in this study did not show significant differences, a cautious approach should still be maintained in future research endeavours.

### Limitations and future research directions

This meta-analysis has identified several limitations and avenues for future research. First, a primary limitation concerns the literature search strategy. This review relied on a set of specific academic databases, and the exclusion of other databases, such as Scopus, may have resulted in the omission of some relevant studies. This decision was primarily based on the significant overlap in coverage between the selected databases and Scopus. Future meta-analyses could benefit from a broader search encompassing multiple databases to further ensure comprehensiveness. Second, twelve leadership styles related to perceived insider status were included in this study. However, due to insufficient research on some leadership styles, they were not included in the meta-analysis. For instance, although authoritarian leadership and benevolent leadership are dimensions of paternalistic leadership,. paternalistic leadership was not included in this meta-analysis due to the lack of studies. Future research could explore more leadership styles to reduce bias in meta-analysis results. And our coding strategy was intentionally conservative, adhering strictly to the labels and definitions provided by the original authors of the primary studies. While this approach enhances reproducibility and minimizes subjective reinterpretation, it may not have fully accounted for the construct overlap between certain styles. Future studies should aim to better disentangle these constructs theoretically and empirically. Third, there are some leadership styles like participative leadership and moral leadership only included few samples, when effect sizes are derived from a small number of studies, the meta-analysis may be affected by random sample selection. Thus, estimates based on small samples should be interpreted carefully. Third, In the relative weight analysis section, there were still missing correlation coefficients between variables, such as the effect sizes of differential leadership and participative leadership, which were not analysed in this study. Future research could address this gap by providing a more comprehensive discussion of the relationship between leadership styles and perceived insider status. At last, future research could further explore additional moderating effects. Such as cultural background differences. Traditionality has been shown to moderate the relationship between perceived insider status and leadership style (Wang et al., 2009; Shen et al., 2020), Chinese traditionality can impact the followers’ response to leadership in the East and the West (Spreitzer et al., 2005). So in collectivist cultures, leadership behaviour may have a more significant impact on subordinates. Additionally, industry characteristics and other factors could potentially influence the relationship between leadership styles and perceived insider status. These factors warrant investigation in future studies.

## Conclusion

This study conducted a meta-analysis on 137 empirical studies, encompassing 151 effect sizes and 45,228 research participants, and found the following results: There is a significant positive correlation between leader-member exchange, differential leadership, inclusive leadership, participative leadership, transformational leadership, empowering leadership, authentic leadership, servant leadership, humble leadership, benevolent leadership, moral leadership and perceived insider status. Authoritarian leadership, however, exhibits a significant negative correlation with perceived insider status. There are differences in the explanatory power of leadership styles on perceived insider status. In descending order of explanatory power, inclusive leadership, empowering leadership, authentic leadership, servant leadership, humble leadership, leader-member exchange, and transformational leadership have diminishing explanatory power on perceived insider status. The moderation effects of study design, measurement tools of leadership, publication status, gender, and age on the relationship between leadership styles and perceived insider status were found to be non-significant.
