# Supplementary material for: The associations between leadership styles and perceived insider status: a meta-analysis
Source: Front Psychol. 2025 Nov 13;16:1631075. doi: 10.3389/fpsyg.2025.1631075 (PMC12657495; doi:10.3389/fpsyg.2025.1631075)
Supplement: Supplementary file 1 [file Table_1.docx]

**Table S1 Leadership definition**

| Leadership | Definition | Measurement Instruments |
| --- | --- | --- |
| Leader-member exchange | LMX refers to the quality of reciprocal exchange relationship between leaders and their followers (Graen & Uhl-Bien, 1995). | 7-item unidimensional LMX scale (Graen & Cashman, 1975); |
| Differential leadership | Differential leadership usually provides more emotional attachment, intimacy, opportunities for promotion and awards to insiders. “Insiders” refer to employees who are close to their leaders and are treated preferentially (Jiang & Zhang, 2010). | 14-items differential leadership scale, which contains three dimensions: communication care, tolerance trust, and promotion reward (Jiang & Zhang, 2010). |
| Inclusive leadership | Inclusive leadership refers to leader behaviors that invite and appreciate input from others and thus help shape team members beliefs that ‘their voices are genuinely valued’ (Nembhard & Edmondson, 2006). | Carmeli et al. (2010) developed a three-dimensional scale for inclusive leadership, including openness, availability, and accessibility. |
| Participative leadership | Participative Leadership involves employees in organizational decision-making, negotiates with team members and share problem-solving solutions before making decisions (Bass, 1990). | six-item scale developed by Arnold et al. (2000). |
| Transformational leadership | Transformational Leadership focuses on inspiring employees' higher-level needs by changing followers' attitudes, beliefs, and values, encouraging them to find meaning in their work (Bass, 1985) | Measured by Multifactor Leadership Questionnaire (MLQ), encompasses four dimensions: charisma, inspiration, intellectual stimulation, and individualized consideration (Bass & Avolio, 1995), |
| Empowering leadership | Empowering leadership is defined as leader behavior directed at individuals or teams that involves delegating authority to employees, promoting their self‐directed and autonomous decision making, coaching, sharing information, and asking for input (Sharma & Kirkman, 2015). | Measured by Empowering Leadership Behaviour Scale, including enhancing the meaningfulness of work, promoting decision involvement, having confidence in subordinates, and providing autonomy (Ahearne et al., 2005). |
| Authentic leadership | Authentic Leadership is a style characterized by behaviours consistent with one's values, emphasizing genuine relationships with subordinates and colleagues and focusing on followers' development (Gardner et al., 2005) | Measured by scales developed by Walumbwa et al. (2008), which include dimensions such as self-awareness, relational transparency, information processing, and internalized morality. |
| Servant leadership | Servant leaders generate followers’ respect by displaying a willingness to sacrifice for others, employing moral and ethical rules, and providing guidance (van Dierendonck, 2011). | Measured by an 18-item scale from van Dierendonck et al. (2017) |
| Humble leadership | Humble leadership is a leadership that involves viewing oneself accurately, providing an appreciation of others’ strengths and contributions, and modeling teachability (Owens et al., 2013). | Measured by a 9-item questionnaire from Owens et al. (2013). |
| Authoritarian leadership | Authoritarian leadership is a unique style among Chinese business leaders, characterized by dictatorship, belittling subordinates, and lecturing (Wang et al., 2010). | Measured by a 13-item scale developed by Cheng, Chou, and Farh (2000). |
| Benevolent leadership | Benevolent Leadership includes personalized care and protection, shows holistic and individualized care for employees’ well-being in both work and non-work domains (Fan & Cheng, 2000). | Measured by an 11-item scale developed by Cheng, Chou, and Farh (2000). |
| Moral leadership | Moral Leadership refers to leaders possessing good moral qualities, emphasizing leading by example, separating public and private matters, and respecting the values of subordinates (Zhou & Long, 2007). | Measured by a 6-item scale developed by Cheng, Chou, and Farh (2000). |

**Table S2 Studies Included in the Meta-analysis**

| Author (Year) | N | Leadership | r | IV | DV | Measurement | type | Study design |
| --- | --- | --- | --- | --- | --- | --- | --- | --- |
| Chen, 2022 | 322 | IL | 0.465 | 0.867 | 0.882 | Carmeli | Unpublished | Cross-sectional |
| Diao et al., 2020 | 203 | IL | 0.460 | 0.914 | 0.817 | Carmeli | Published | Cross-sectional |
| Ding et al., 2017 | 418 | IL | 0.208 | 0.908 | 0.789 | Other | Published | Cross-sectional |
| Guo, 2021 | 326 | IL | 0.430 | 0.925 | 0.826 | Carmeli | Published | Cross-sectional |
| Li & Peng, 2023 | 220 | IL | 0.569 | 0.896 | 0.889 | Carmeli | Published | Time-lagged |
| Lin, 2018 | 327 | IL | 0.389 | 0.937 | 0.898 | Carmeli | Unpublished | Cross-sectional |
| Liu, 2019 | 367 | IL | 0.806 | 0.917 | 0.860 | Carmeli | Unpublished | Cross-sectional |
| Naseer et al., 2023 | 300 | IL | 0.310 | 0.840 | 0.820 | Carmeli | Published | Time-lagged |
| Ren et al., 2023 | 173 | IL | 0.585 | 0.808 | 0.811 | Other | Published | Cross-sectional |
| Shen, 2020 | 398 | IL | 0.519 | 0.898 | 0.892 | Carmeli | Unpublished | Cross-sectional |
| Wang et al., 2018 | 306 | IL | 0.169 | 0.78 | 0.77 | Carmeli | Published | Time-lagged |
| Xin, 2022 | 320 | IL | 0.687 | 0.917 | 0.942 | Carmeli | Unpublished | Cross-sectional |
| Zeng, 2018 | 251 | IL | 0.673 | 0.943 | 0.906 | Carmeli | Unpublished | Cross-sectional |
| Zhang, Y, Q., 2022 | 386 | IL | 0.606 | 0.859 | 0.902 | Carmeli | Published | Cross-sectional |
| Zhang, Y, R., 2022 | 535 | IL | 0.655 | 0.909 | 0.87 | Carmeli | Unpublished | Cross-sectional |
| Zhao et al., 2020 | 458 | IL | 0.711 | 0.837 | 0.855 | Other | Published | Cross-sectional |
| Zhong et al., 2020 | 185 | IL | 0.570 | 0.920 | 0.870 | Carmeli | Published | Time-lagged |
| Deng, 2020 | 241 | DL | 0.07 | 0.859 | 0.868 | Jiang | Unpublished | Cross-sectional |
| Gao, 2015 | 511 | DL | 0.838 | 0.91 | 0.85 | Jiang | Unpublished | Cross-sectional |
| Huo et al., 2023 | 92 | DL | -0.065 | 0.936 | 0.751 | Jiang | Published | Time-lagged |
| Jiang et al., 2023 | 546 | DL | 0.270 | 0.880 | 0.850 | Jiang | Published | Cross-sectional |
| Li, 2019 | 364 | DL | 0.526 | 0.879 | 0.905 | Jiang | Unpublished | Cross-sectional |
| Li et al., 2019 | 511 | DL | 0.840 | 0.910 | 0.850 | Jiang | Published | Cross-sectional |
| Liu, 2020 | 380 | DL | 0.050 | 0.903 | 0.892 | Jiang | Unpublished | Cross-sectional |
| Liu, 2022 | 396 | DL | 0.351 | 0.946 | 0.915 | Jiang | Unpublished | Cross-sectional |
| Ma & Bai, 2022 | 878 | DL | 0.577 | 0.947 | 0.876 | Jiang | Published | Time-lagged |
| Shu, 2021 | 330 | DL | 0.351 | 0.878 | 0.824 | Jiang | Unpublished | Cross-sectional |
| Sun & Lu, 2019 | 168 | DL | 0.221 | 0.862 | 0.892 | Jiang | Published | Cross-sectional |
| Wang, 2019 | 288 | DL | 0.599 | 0.926 | 0.836 | Jiang | Unpublished | Cross-sectional |
| Xie, 2019 | 402 | DL | 0.349 | 0.899 | 0.855 | Jiang | Unpublished | Cross-sectional |
| Yang, 2021 | 367 | DL | 0.608 | 0.892 | 0.873 | Jiang | Unpublished | Cross-sectional |
| Zhang, 2014 | 651 | DL | 0.328 | 0.916 | 0.867 | Jiang | Unpublished | Cross-sectional |
| Zhang, 2019 | 332 | DL | 0.144 | 0.939 | 0.849 | Jiang | Unpublished | Cross-sectional |
| Zhang, 2021 | 397 | DL | 0.286 | 0.903 | 0.86 | Jiang | Unpublished | Cross-sectional |
| Zhang et al., 2022 | 332 | DL | 0.144 | 0.939 | 0.849 | Jiang | Published | Cross-sectional |
| Zhao, 2019 | 394 | DL | 0.170 | 0.860 | 0.910 | Jiang | Published | Cross-sectional |
| Zhao, 2023 | 434 | DL | 0.617 | 0.930 | 0.933 | Jiang | Unpublished | Cross-sectional |
| Han, 2022 | 580 | AL | 0.516 | 0.969 | 0.969 | Walumbwa | Unpublished | Cross-sectional |
| Hou, 2021 | 293 | AL | 0.561 | 0.731 | 0.701 | Walumbwa | Unpublished | Cross-sectional |
| Li et al., 2021 | 418 | AL | 0.518 | 0.886 | 0.888 | Walumbwa | Published | Cross-sectional |
| Wang et Zhang, 2019 | 303 | AL | 0.357 | 0.930 | 0.877 | Walumbwa | Published | Cross-sectional |
| Zhao et al., 2021 | 336 | AL | 0.450 | 0.940 | 0.840 | Walumbwa | Published | Time-lagged |
| Zhao et Jiang, 2017 | 296 | AL | 0.480 | 0.900 | 0.830 | Walumbwa | Published | Cross-sectional |
| Chen et al., 2023 | 224 | EL | 0.039 | 0.856 | 0.700 | Other | Published | Time-lagged |
| Deng, 2021 | 188 | EL | 0.340 | 0.850 | 0.753 | Ahearne | Published | Cross-sectional |
| Geng, 2016 | 210 | EL | 0.417 | 0.873 | 0.684 | Other | Unpublished | Cross-sectional |
| Han, 2021 | 218 | EL | 0.417 | 0.902 | 0.706 | Other | Unpublished | Cross-sectional |
| Li, 2019 | 296 | EL | 0.175 | 0.778 | 0.907 | Ahearne | Unpublished | Cross-sectional |
| Li, 2020 | 325 | EL | 0.387 | 0.944 | 0.937 | Ahearne | Unpublished | Cross-sectional |
| Sang, 2021 | 284 | EL | 0.740 | 0.954 | 0.859 | Ahearne | Unpublished | Cross-sectional |
| Wang, 2020 | 460 | EL | 0.540 | 0.882 | 0.858 | Ahearne | Unpublished | Cross-sectional |
| Wang, 2022 | 353 | EL | 0.461 | 0.935 | 0.956 | Ahearne | Unpublished | Cross-sectional |
| Wang et al., 2019 | 276 | EL | 0.595 | 0.933 | 0.935 | Ahearne | Published | Cross-sectional |
| Wei & Hao, 2023 | 220 | EL | 0.634 | 0.931 | 0.916 | Ahearne | Published | Cross-sectional |
| Yang &Yang, 2021 | 350 | EL | 0.640 | 0.894 | 0.841 | Ahearne | Published | Cross-sectional |
| Zhang, 2023 | 407 | EL | 0.422 | 0.914 | 0.86 | Ahearne | Unpublished | Cross-sectional |
| Zhang, 2022 | 435 | EL | 0.662 | 0.919 | 0.849 | Ahearne | Unpublished | Cross-sectional |
| Zhang et al., 2022 | 376 | EL | 0.623 | 0.932 | 0.86 | Ahearne | Published | Cross-sectional |
| Zhao & Yan, 2016 | 333 | EL | 0. 480 | 0.840 | 0.840 | Ahearne | Published | Time-lagged |
| Kang et al., 2019 | 190 | AUL | -0.450 | 0.830 | 0.870 | Cheng | Published | Time-lagged |
| Qin, 2021 | 326 | AUL | -0.256 | 0.940 | 0.866 | Cheng | Unpublished | Cross-sectional |
| Schaubroeck et al., 2017 | 202 | AUL | -0.150 | 0.900 | 0.820 | Cheng | Published | Cross-sectional |
| Shen et al., 2020 | 315 | AUL | 0.136 | 0.790 | 0.880 | Cheng | Published | Cross-sectional |
| Wang et al., 2010 | 267 | AUL | -0.120 | 0.880 | 0.840 | Cheng | Published | Cross-sectional |
| Wu, 2019 | 453 | AUL | -0.258 | 0.734 | 0.809 | Cheng | Unpublished | Cross-sectional |
| Xi, 2020 | 599 | AUL | -0.380 | 0.820 | 0.770 | Cheng | Unpublished | Cross-sectional |
| Xing, 2022 | 253 | AUL | -0.240 | 0.770 | 0.840 | Cheng | Published | Cross-sectional |
| Xu, 2020 | 358 | AUL | -0.225 | 0.841 | 0.911 | Cheng | Unpublished | Cross-sectional |
| Zhang, 2018 | 223 | AUL | -0.242 | 0.827 | 0.837 | Cheng | Published | Cross-sectional |
| Zhang et al., 2021 | 286 | AUL | 0.160 | 0.831 | 0.838 | Cheng | Published | Cross-sectional |
| Liu, 2019 | 244 | BL | 0.432 | 0.825 | 0.864 | Cheng | Unpublished | Time-lagged |
| Qin, 2021 | 326 | BL | 0.351 | 0.910 | 0.866 | Cheng | Unpublished | Cross-sectional |
| Shen et al., 2017 | 215 | BL | 0.30 | 0.85 | 0.80 | Cheng | Published | Time-lagged |
| Shen et al., 2020 | 315 | BL | 0.340 | 0.870 | 0.880 | Cheng | Published | Cross-sectional |
| Wei, 2020 | 348 | BL | 0.510 | 0.940 | 0.905 | Cheng | Unpublished | Cross-sectional |
| Wu, 2019 | 453 | BL | 0.436 | 0.877 | 0.809 | Cheng | Unpublished | Cross-sectional |
| Xi, 2020 | 599 | BL | 0.400 | 0.920 | 0.770 | Cheng | Unpublished | Cross-sectional |
| Xu, 2020 | 358 | BL | 0.430 | 0.794 | 0.911 | Cheng | Unpublished | Cross-sectional |
| Zhang, 2018 | 223 | BL | 0.493 | 0.880 | 0.844 | Cheng | Published | Cross-sectional |
| Wu, 2019 | 453 | ML | 0.537 | 0.870 | 0.809 | Cheng | Unpublished | Cross-sectional |
| Xi, 2020 | 599 | ML | 0.500 | 0.930 | 0.770 | Cheng | Unpublished | Cross-sectional |
| Xu, 2020 | 358 | ML | 0.426 | 0.791 | 0.911 | Cheng | Unpublished | Cross-sectional |
| Zhang, 2018 | 223 | ML | 0.416 | 0.880 | 0.818 | Cheng | Published | Cross-sectional |
| Hao, 2020 | 269 | SL | 0.480 | 0.961 | 0.904 | Other | Unpublished | Cross-sectional |
| Jiang, 2016 | 340 | SL | 0.509 | 0.964 | 0.867 | Other | Unpublished | Cross-sectional |
| Liao, 2021 | 367 | SL | 0.454 | 0.954 | 0.899 | Ehrhart | Unpublished | Cross-sectional |
| Opoku et al., 2019 | 213 | SL | 0.420 | 0.880 | 0.860 | Other | Published | Time-lagged |
| Qu, 2023 | 322 | SL | 0.761 | 0.938 | 0.911 | Ehrhart | Unpublished | Cross-sectional |
| Xu, 2018 | 316 | SL | 0.552 | 0.969 | 0.945 | Ehrhart | Unpublished | Cross-sectional |
| Xiao, 2021 | 206 | SL | 0.656 | 0.913 | 0.827 | Ehrhart | Unpublished | Cross-sectional |
| Yeh et al., 2022 | 285 | SL | 0.480 | 0.930 | 0.840 | Other | Published | Cross-sectional |
| Zeng & Xu, 2020 | 269 | SL | 0.470 | 0.950 | 0.900 | Other | Published | Time-lagged |
| Zhou, 2022 | 371 | SL | 0.317 | 0.938 | 0.893 | Other | Unpublished | Cross-sectional |
| Chen, 2014 | 268 | LMX | 0.289 | 0.906 | 0.77 | Wang | Unpublished | Cross-sectional |
| Guo, 2021 | 326 | LMX | 0.477 | 0.862 | 0.826 | Graen | Published | Cross-sectional |
| Jiang, 2017 | 368 | LMX | 0.370 | 0.941 | 0.828 | Wang | Unpublished | Cross-sectional |
| Jiang et al., 2019 | 328 | LMX | 0.238 | 0.960 | 0.760 | Wang | Published | Cross-sectional |
| Li et al., 2014 | 283 | LMX | 0.190 | 0.870 | 0.820 | Graen | Published | Cross-sectional |
| Liao, 2014 | 200 | LMX | 0.471 | 0.909 | 0.832 | Wang | Unpublished | Cross-sectional |
| Niu et al., 2022 | 473 | LMX | 0.069 | 0.877 | 0.858 | Other | Published | Time-lagged |
| Ou et al., 2018 | 320 | LMX | 0.360 | 0.880 | 0.780 | Graen | Published | Cross-sectional |
| Qian et al., 2015 | 254 | LMX | 0.532 | 0.935 | 0.938 | Wang | Published | Cross-sectional |
| Shi et al., 2019 | 444 | LMX | 0.410 | 0.795 | 0.842 | Graen | Published | Cross-sectional |
| Shi & Guo, 2021 | 183 | LMX | 0.30 | 0.920 | 0.890 | Graen | Published | Time-lagged |
| Shen et al., 2017 | 215 | LMX | 0.460 | 0.800 | 0.860 | Graen | Published | Cross-sectional |
| Sui & Wang, 2014 | 183 | LMX | 0.490 | 0.880 | 0.880 | Other | Published | Cross-sectional |
| Tang & Zhao, 2018 | 310 | LMX | 0.440 | 0.880 | 0.910 | Graen | Published | Cross-sectional |
| Wang, 2013 | 218 | LMX | 0.524 | 0.834 | 0.892 | Graen | Unpublished | Cross-sectional |
| Wang, 2022 | 305 | LMX | -0.368 | 0.877 | 0.858 | Wang | Unpublished | Cross-sectional |
| Wang & Zhang, 2018 | 188 | LMX | 0.323 | 0.876 | 0.845 | Graen | Published | Cross-sectional |
| Wang et al., 2009 | 214 | LMX | 0.454 | 0.862 | 0.871 | Graen | Published | Cross-sectional |
| Wang et al., 2019 | 186 | LMX | 0.560 | 0.830 | 0.830 | Graen | Published | Cross-sectional |
| Wang et al., 2022 | 405 | LMX | 0.518 | 0.857 | 0.881 | Graen | Published | Cross-sectional |
| Wu, 2020 | 936 | LMX | 0.623 | 0.819 | 0.801 | Graen | Unpublished | Cross-sectional |
| Xiao, 2010 | 253 | LMX | 0.488 | 0.774 | 0.766 | Graen | Unpublished | Cross-sectional |
| Xie et al., 2014 | 234 | LMX | 0.430 | 0.797 | 0.775 | Other | Published | Cross-sectional |
| Xia, 2022 | 787 | LMX | 0.110 | 0.910 | 0.943 | Other | Unpublished | Time-lagged |
| Xu, 2018 | 316 | LMX | 0.624 | 0.954 | 0.945 | Other | Unpublished | Time-lagged |
| Yang et al., 2015 | 308 | LMX | 0.530 | 0.890 | 0.820 | Graen | Published | Cross-sectional |
| Yang, 2018 | 300 | LMX | 0.470 | 0.945 | 0.835 | Other | Unpublished | Cross-sectional |
| Yin & Huang, 2011 | 190 | LMX | 0.533 | 0.875 | 0.856 | Other | Published | Cross-sectional |
| Yuan, 2016 | 635 | LMX | 0.394 | 0.898 | 0.912 | Graen | Unpublished | Cross-sectional |
| Zeng & Xu, 2020 | 269 | LMX | 0.420 | 0.880 | 0.900 | Graen | Published | Time-lagged |
| Zhang et al., 2016 | 176 | LMX | 0.550 | 0.820 | 0.820 | Graen | Published | Cross-sectional |
| Zhang, 2019 | 206 | LMX | 0.470 | 0.877 | 0.858 | Other | Unpublished | Cross-sectional |
| Zhao et al., 2014 | 358 | LMX | 0.180 | 0.860 | 0.950 | Graen | Published | Cross-sectional |
| Zhong et al., 2015 | 200 | LMX | 0.471 | 0.905 | 0.832 | Wang | Published | Cross-sectional |
| Zhou, 2022 | 359 | LMX | 0.225 | 0.941 | 0.919 | Other | Unpublished | Time-lagged |
| Dong, 2019 | 199 | HL | 0.480 | 0.961 | 0.825 | Owens | Unpublished | Time-lagged |
| Jia et al., 2023 | 308 | HL | 0.308 | 0.945 | 0.817 | Owens | Unpublished | Time-lagged |
| Ni & Du, 2017 | 208 | HL | 0.443 | 0.826 | 0.877 | Owens | Published | Cross-sectional |
| Shi, 2022 | 384 | HL | 0.476 | 0.887 | 0.867 | Owens | Unpublished | Cross-sectional |
| Su et al., 2017 | 326 | HL | 0.823 | 0.940 | 0.885 | Owens | Published | Cross-sectional |
| Wang & Luo, 2017 | 546 | HL | 0. 450 | 0.880 | 0.850 | Owens | Published | Cross-sectional |
| Wang & Zhang, 2018 | 188 | HL | 0.316 | 0.869 | 0.845 | Owens | Published | Cross-sectional |
| Wei, 2018 | 287 | HL | 0.538 | 0.925 | 0.875 | Owens | Unpublished | Cross-sectional |
| Yang, 2020 | 372 | HL | 0.233 | 0.951 | 0.938 | Owens | Unpublished | Cross-sectional |
| Yang, 2021 | 373 | HL | 0.304 | 0.900 | 0.931 | Owens | Unpublished | Cross-sectional |
| Zhou, 2016 | 147 | HL | 0.609 | 0.885 | 0.876 | Owens | Unpublished | Cross-sectional |
| Zhu, Wu, & Song, 2019 | 650 | HL | 0.510 | 0.890 | 0.850 | Owens | Published | Cross-sectional |
| Zhu, Zhang, & Shen, 2019 | 434 | HL | 0.510 | 0.900 | 0.880 | Owens | Published | Cross-sectional |
| Irangani et al., 2021 | 426 | TL | 0.268 | 0.813 | 0.904 | Other | Published | Cross-sectional |
| Liu, 2017 | 314 | TL | 0.129 | 0.956 | 0.938 | Li | Unpublished | Cross-sectional |
| Orynbayeva Assel, 2021 | 300 | TL | 0.150 | 0.944 | 0.701 | Bass | Unpublished | Cross-sectional |
| Su & Xu, 2019 | 251 | TL | 0.569 | 0.933 | 0.890 | Bass | Published | Cross-sectional |
| Tan, 2021 | 454 | TL | 0.522 | 0.850 | 0.761 | Bass | Unpublished | Cross-sectional |
| Wang, 2021 | 365 | TL | 0.463 | 0.983 | 0.955 | Li | Unpublished | Cross-sectional |
| Dou & Shen, 2021 | 282 | PL | 0.440 | 0.950 | 0.860 | Arnold | Published | Time-lagged |
| Fan, 2018 | 353 | PL | 0.169 | 0.771 | 0.824 | Arnold | Unpublished | Time-lagged |
| Men, 2018 | 204 | PL | 0.271 | 0.721 | 0.792 | Arnold | Unpublished | Time-lagged |
| Rose, 2018 | 303 | PL | 0.398 | 0.790 | 0.810 | Arnold | Published | Time-lagged |

**Table S3 Moderator analysis results for categorical variables**

| Moderator | Variable | Type | k | ρ | 95% CI | Z-value | Q_W_ | Q_b_ | p |
| --- | --- | --- | --- | --- | --- | --- | --- | --- | --- |
| Study design | LMX | Time-lagged | 6 | 0.38 | [0.16, 0.57] | 3.32 | 159.47 | 0.83 | 0.36 |
|  |  | Cross-sectional | 28 | 0.48 | [0.39, 0.56] | 9.31 | 729.18 |  |  |
|  | IL | Time-lagged | 4 | 0.49 | [0.19, 0.70] | 3.08 | 53.24 | 1.89 | 0.17 |
|  |  | Cross-sectional | 13 | 0.67 | [0.55, 0.76] | 8.41 | 514.76 |  |  |
| Measurement | LMX | Graen | 18 | 0.52 | [0.41, 0.61] | 8.43 | 327.84 | 2.93 | 0.23 |
|  |  | Wang | 7 | 0.34 | [0.14, 0.52] | 3.25 | 249.24 |  |  |
|  |  | Other | 9 | 0.45 | [0.29, 0.59] | 5.06 | 225.04 |  |  |
|  | IL | Carmeli | 14 | 0.62 | [0.49, 0.73] | 7.28 | 449.22 | 0.08 | 0.78 |
|  |  | Other | 3 | 0.66 | [0.36, 0.84] | 3.69 | 205.51 |  |  |
|  | EL | Ahearne | 13 | 0.60 | [0.51, 0.69] | 9.63 | 255.42 | 2.93 | 0.09 |
|  |  | Other | 3 | 0.39 | [0.11, 0.61] | 2.68 | 42.37 |  |  |
|  | SL | Ehrhart | 4 | 0.67 | [0.53, 0.77] | 7.50 | 86.93 | 3.19 | 0.07 |
|  |  | Other | 6 | 0.51 | [0.37, 0.62] | 6.38 | 19.79 |  |  |
| Publication Type | LMX | published | 22 | 0.49 | [0.38, 0.58] | 7.95 | 257.98 | 0.41 | 0.52 |
|  |  | Unpublished | 12 | 0.43 | [0.28, 0.56] | 5.10 | 715.43 |  |  |
|  | DL | published | 7 | 0.47 | [0.08, 0.73] | 2.33 | 1150.19 | 0.04 | 0.85 |
|  |  | Unpublished | 13 | 0.51 | [0.24, 0.70] | 3.50 | 1158.05 |  |  |
|  | IL | published | 11 | 0.59 | [0.43, 0.71] | 6.24 | 341.95 | 1.00 | 0.32 |
|  |  | Unpublished | 6 | 0.69 | [0.52, 0.82] | 5.87 | 262.65 |  |  |
|  | EL | published | 7 | 0.58 | [0.42, 0.70] | 6.05 | 143.88 | 0.01 | 0.91 |
|  |  | Unpublished | 9 | 0.56 | [0.42, 0.68] | 6.71 | 203.18 |  |  |
|  | SL | published | 3 | 0.51 | [0.28, 0.69] | 3.99 | 0.80 | 0.63 | 0.43 |
|  |  | Unpublished | 7 | 0.60 | [0.48, 0.71] | 7.59 | 138.37 |  |  |
|  | AUL | published | 7 | -0.16 | [-0.31, 0.00] | -1.95 | 110.17 | 2.01 | 0.16 |
|  |  | Unpublished | 4 | -0.34 | [-0.51, -0.14] | -3.29 | 20.16 |  |  |
|  | BL | published | 3 | 0.44 | [0.36, 0.52] | 8.96 | 9.83 | 1.19 | 0.28 |
|  |  | Unpublished | 6 | 0.50 | [0.44, 0.55] | 15.85 | 7.53 |  |  |
|  | HL | published | 7 | 0.56 | [0.39, 0.70] | 5.43 | 317.01 | 0.05 | 0.82 |
|  |  | Unpublished | 6 | 0.54 | [0.33, 0.69] | 4.69 | 32.12 |  |  |

**Table S4 Moderator analysis results for continuous variables**

| Moderator | Variable | k | B | SE | 95% CI | Z-value | p |
| --- | --- | --- | --- | --- | --- | --- | --- |
| Gender | LMX | 33 | 0.59 | 0.53 | [-0.44, 1.63] | 1.13 | 0.26 |
|  | DL | 19 | 0.38 | 1.84 | [-3.23, 3.98] | 0.20 | 0.84 |
|  | IL | 16 | 0.35 | 0.67 | [-0.96, 1.65] | 0.52 | 0.60 |
|  | EL | 15 | -0.30 | 0.50 | [-1.28, 0.68] | -0.60 | 0.55 |
|  | SL | 10 | 0.36 | 0.91 | [-1.42, 2.14] | 0.39 | 0.70 |
|  | HL | 13 | 0.28 | 0.52 | [-0.75, 1.31] | 0.54 | 0.59 |
| Age | LMX | 29 | -0.01 | 0.01 | [-0.03, 0.02] | -0.51 | 0.61 |
|  | DL | 17 | 0.00 | 0.03 | [-0.05, 0.05] | 0.02 | 0.98 |
|  | IL | 16 | -0.03 | 0.03 | [-0.09, 0.03] | -0.98 | 0.33 |
|  | EL | 14 | -0.00 | 0.03 | [-0.05, 0.05] | -0.07 | 0.94 |
|  | SL | 10 | -0.01 | 0.02 | [-0.05, 0.02] | -0.69 | 0.49 |
|  | HL | 11 | 0.00 | 0.01 | [-0.02, 0.02] | 0.15 | 0.88 |
